# Impact of admixture and ancestry on eQTL analysis and GWAS colocalization in GTEx

**DOI:** 10.1186/s13059-020-02113-0

**Published:** 2020-09-11

**Authors:** Nicole R. Gay, Michael Gloudemans, Margaret L. Antonio, Nathan S. Abell, Brunilda Balliu, YoSon Park, Alicia R. Martin, Shaila Musharoff, Abhiram S. Rao, François Aguet, Alvaro N. Barbeira, Rodrigo Bonazzola, Farhad Hormozdiari, GTEx Consortium, Kristin G. Ardlie, Christopher D. Brown, Hae Kyung Im, Tuuli Lappalainen, Xiaoquan Wen, Stephen B. Montgomery

**Affiliations:** 1 Department of Genetics, Stanford University, Stanford, CA USA; 2 Biomedical Informatics, Stanford University, Stanford, CA USA; 3 Department of Biomathematics, University of California, Los Angeles, Los Angeles, CA USA; 4 Department of Genetics, Perelman School of Medicine, University of Pennsylvania, Philadelphia, PA USA; 5 Department of Systems Pharmacology and Translational Therapeutics, Perelman School of Medicine, University of Pennsylvania, Philadelphia, PA USA; 6 Analytic and Translational Genetics Unit, Massachusetts General Hospital, Boston, MA USA; 7 Stanley Center for Psychiatric Research, Broad Institute, Cambridge, MA USA; 8 Department of Bioengineering, Stanford University, Stanford, CA USA; 9 The Broad Institute of MIT and Harvard, Cambridge, MA USA; 10 Section of Genetic Medicine, Department of Medicine, The University of Chicago, Chicago, IL USA; 11 Department of Epidemiology, Harvard T.H. Chan School of Public Health, Boston, MA USA; 12 New York Genome Center, New York, NY USA; 13 Department of Systems Biology, Columbia University, New York, NY USA; 14 Department of Biostatistics, University of Michigan, Ann Arbor, MI USA; 15 Department of Pathology, Stanford University, Stanford, CA USA

**Keywords:** Local ancestry, Population structure, Admixture, eQTL, Colocalization, GTEx, Gene expression

## Abstract

**Background:**

Population structure among study subjects may confound genetic association studies, and lack of proper correction can lead to spurious findings. The Genotype-Tissue Expression (GTEx) project largely contains individuals of European ancestry, but the v8 release also includes up to 15% of individuals of non-European ancestry. Assessing ancestry-based adjustments in GTEx improves portability of this research across populations and further characterizes the impact of population structure on GWAS colocalization.

**Results:**

Here, we identify a subset of 117 individuals in GTEx (v8) with a high degree of population admixture and estimate genome-wide local ancestry. We perform genome-wide *cis*-eQTL mapping using admixed samples in seven tissues, adjusted by either global or local ancestry. Consistent with previous work, we observe improved power with local ancestry adjustment. At loci where the two adjustments produce different lead variants, we observe 31 loci (0.02%) where a significant colocalization is called only with one eQTL ancestry adjustment method. Notably, both adjustments produce similar numbers of significant colocalizations within each of two different colocalization methods, COLOC and FINEMAP. Finally, we identify a small subset of eQTL-associated variants highly correlated with local ancestry, providing a resource to enhance functional follow-up.

**Conclusions:**

We provide a local ancestry map for admixed individuals in the GTEx v8 release and describe the impact of ancestry and admixture on gene expression, eQTLs, and GWAS colocalization. While the majority of the results are concordant between local and global ancestry-based adjustments, we identify distinct advantages and disadvantages to each approach.

## Introduction

Thousands of genome-wide association studies (GWAS) have been published to date. Subsequently, large-scale expression quantitative trait loci (eQTL) datasets are studied to provide insights for genetic variants associated with complex traits. While the majority of such studies focus on single-ancestry populations or relatively homogeneous populations, the latest Genotype-Tissue Expression (GTEx) project (v8) includes up to 17% of individuals with non-European or admixed ancestry [1]. Genetic studies with individuals of admixed ancestries may suffer from additional challenges due to complex population substructure [2, 3]. Such substructure can confound genetic associations, and insufficient control may increase spurious findings [4, 5].

Global ancestry (GA), or the proportions of different ancestral populations represented across the entire genome, is routinely used to adjust for population structure in genetic association studies [6]. This approach has the advantage of averaging genomic background effects and was used in eQTL mapping for the main GTEx releases [1, 7]. The potential disadvantage of correcting only for GA is that it does not precisely account for ancestry at any specific locus. This can be problematic when genes are differentially expressed in ancestral populations of admixed individuals. In contrast, local ancestry (LA), or the number of alleles derived from distinct ancestral populations at a given locus, may be more appropriate for population structure adjustment in admixed populations but typically suffers from much longer compute time and can be prone to errors in estimation at a variant level [5, 8–12].

LA adjustment in genetic association studies has been shown to reduce type I error rate (false positives) [13–15] and sufficiently control for population stratification [13, 15]. However, the power of adjusting for LA is highly dependent on the underlying genetic architecture of the admixed population [8, 12, 15–17]; some have recommended using LA adjustment as a method for follow-up of candidate loci as opposed to a discovery tool for GWAS [8, 14, 18]. Fewer studies have investigated the effect of LA adjustment on eQTL mapping, demonstrating modest improvements in discovery power [5, 10]. Recently, Zhong et al. have demonstrated that the use of LA adjustment, compared to GA adjustment, can improve eQTL mapping while controlling for type I error rate and increasing statistical power [10]. However, the implications of these differences for GWAS colocalization were not assessed.

In this study, we describe the degree of admixture in the GTEx v8 cohort and estimate LA for a subset of 117 individuals with at least 10% admixture from European, African, and East Asian ancestral populations. LA explains at least 7% of the variance in residual expression for 1% of expressed genes (*M* = 1159). We perform *cis*-eQTL mapping in seven tissues and assess the differences between LA adjustment and GA adjustment in the context of this admixed sub-cohort. For the subset of loci where the two ancestry adjustment methods yield different results, we perform GWAS/eQTL colocalization analyses with 142 previously published GWAS, representing a range of traits, consortia, and cohort ancestry. We characterize 31 loci where a significant colocalization is reported only with one eQTL ancestry adjustment method. Finally, we identify a small subset of GTEx eVariants whose genotypes are highly correlated with LA, providing a resource to enhance functional follow-up of these loci.

## Results

### GTEx includes African and Asian population admixture

**Fig. 1:**
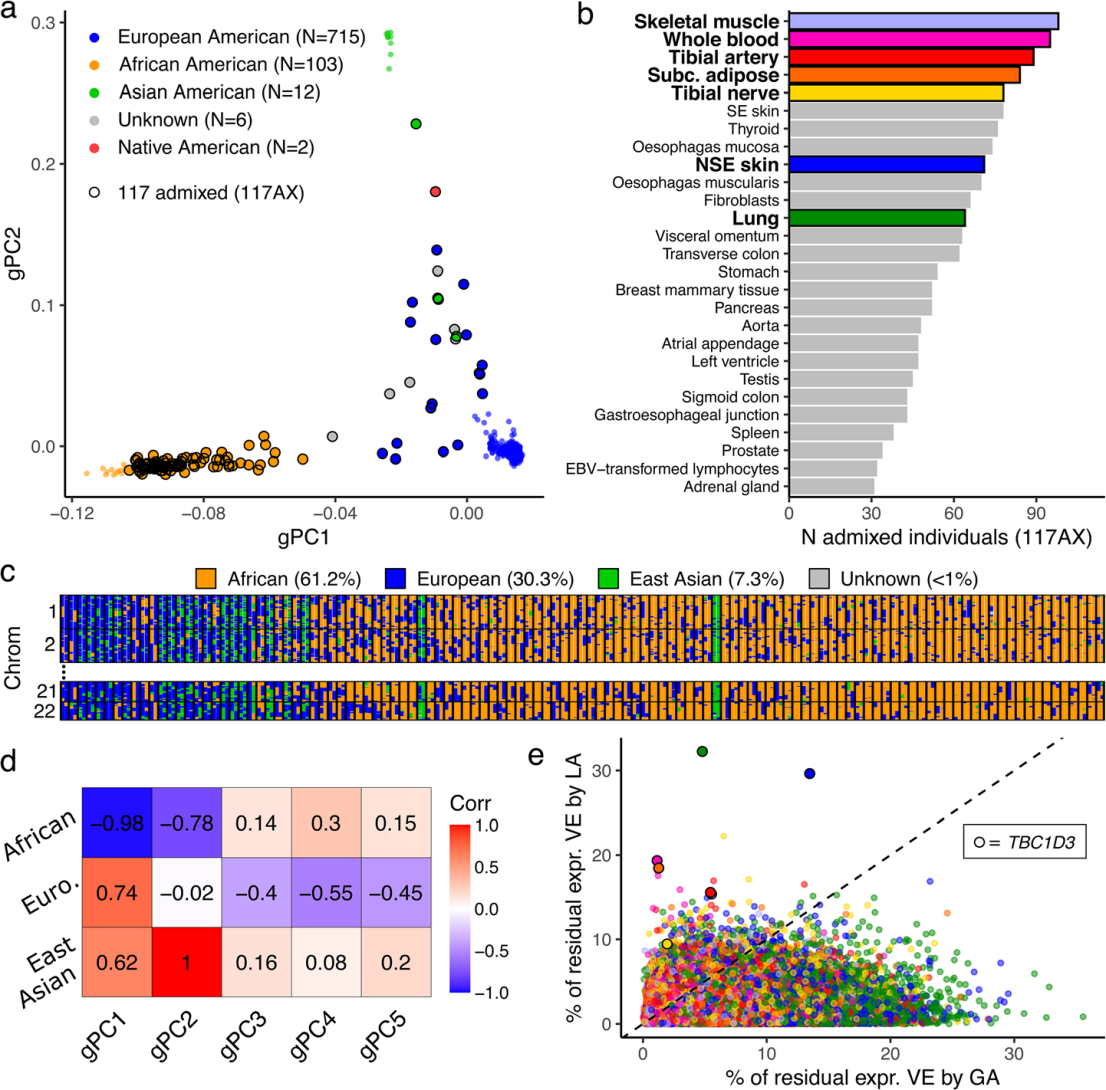
Population admixture in the GTEx v8 cohort. **a** Genotype principal components (gPCs) reflect global ancestry. Points are colored by self-reported ancestry. Circled points indicate the 117 individuals defined as admixed (117AX). **b** A subset of GTEx v8 tissues has an 117AX sample size of at least 30. The seven tissues selected for *cis*-eQTL mapping in 117AX are colored and shown in bold. **c** LA tracts collapse consecutive variants on a single parental chromosome with the same ancestry assignment into contiguous haplotype blocks. The fine spatial resolution of local ancestry contrasts with the global ancestry proportions indicated in the legend. Haplotypes (columns) are paired by individuals; rows are autosomal chromosomes. Individuals are sorted from left to right by decreasing proportions of European admixture. **d** gPCs are highly correlated with global ancestry proportions averaged from genome-wide local ancestry. **e** Local (or global) ancestry explains a fraction of variance in residual gene expression after correcting for global (or local) ancestry. Local ancestry is defined as the local ancestry at the transcription start site of each gene; global ancestry is the first five gPCs. Points are colored by tissue; colors correspond with **b**. Subc., subcutaneous; NSE, not sun-exposed; VE, variance explained; LA, local ancestry; GA, global ancestry

The GTEx v8 release includes whole genome sequencing and gene expression data for 838 individuals, including 103 African American and 12 Asian American individuals (self-reported ancestry). Genome-wide genotype-based principal components (gPCs) reflect GA and have been used to adjust for population structure in both GWAS [6, 9, 13] and eQTL studies [7]. Therefore, to understand the degree of population admixture represented in GTEx, we compared the first two gPCs with self-reported ancestry (Fig. 1a). Figure 1a demonstrates that gPC1 and gPC2 reflect African and Asian ancestry, respectively; the majority of European Americans (698 out of 715 individuals) cluster together near the origin, suggesting that the samples in this cluster are relatively homogeneously European-descendent. These patterns are observed with finer resolution when genotype PCA is performed with combined GTEx and 1000 Genomes data [19] (Additional file 1, Figure S1). A subset of 117 individuals with more than 10% population admixture, referred to as 117AX, was retained for downstream analyses (Fig. 1a; Additional file 2, Table S1).

The 49 tissues used for QTL discovery in the GTEx v8 release have a varying representation of 117AX. Twenty-seven of these tissues have a sample size of at least 30 admixed individuals (Fig. 1b). Sample sizes for all 49 tissues are provided in Figure S2 (Additional file 1). The pituitary and 13 central nervous system tissues have the lowest representation of 117AX relative to total sample sizes per tissue (mean 7%). We selected seven tissues in which to perform *cis*-eQTL calling based on a minimum admixed sample size of 60 [20] and relevance to phenotypes with known population differences (e.g., subcutaneous adipose and body fat distribution [21, 22], *N* = 84; not-sun-exposed (NSE) skin and epidermal gene expression [23], *N* = 71; lung and asthma prevalence [24], *N* = 64; skeletal muscle and lean muscle mass [25], *N* = 98). Whole blood (*N* = 95) and tibial artery (*N* = 89) were also included because they have large 117AX sample sizes.

Using RFMix [26], we performed three-population (European, African, and East Asian) LA estimation on 117AX (see the “Methods” section; Fig. 1c; Additional file 1, Figure S3). We provide these LA calls as a resource for further investigation of GTEx data (Additional file 3, Table S2). For each individual, genome-wide LA was averaged to provide GA estimates. Every sample in 117AX has less than 90% GA from any one ancestral population out of Europe, Africa, and East Asia. We correlated these GA proportions with the first five gPCs, which quantitatively demonstrates the strong relationships between gPC1 and African ancestry (*r* = − 0.98) and gPC2 and East Asian ancestry (*r* = 1.0; Fig. 1d).

In order to assess the importance of LA in the context of gene expression, we adapted an existing approach [27] to calculate the proportion of variance explained in 117AX gene expression by LA after accounting for GA and vice versa (see the “Methods” section; Fig. 1e; Additional file 4, Table S3). On average, across genes in our seven tissues of interest, GA explains more variance in gene expression than LA at the transcription start site for each gene (*P* value < 2.2e−16, two-sided *t* test). However, LA explains at least 7% of the variance in residual expression for 1% of expressed genes (*M* = 1159). At the extreme, LA explains 32% of the variance in residualized expression of *TBC1 domain family member 3 (TBC1D3)*, a hominoid-specific oncogene [28], in the lung; LA also explains significantly more variance in *TBC1D3* expression than GA in all seven tissues tested (*P* value = 0.0018, two-sided *t* test). In a separate study of copy number, *TBC1D3* was among the most variable (median 38.13, variance 93.2 copies among 159 individuals) and population-stratified (mean 29.28, 34.17, and 43.86 copy numbers in European, Asian, and Yoruban samples, respectively) human gene families [29]. Such biological evidence for residual variance in gene expression captured by LA supports the importance of considering LA in the context of eQTL mapping.

### Local ancestry adjustment increases power for discovery in *cis*-eQTL mapping

We performed *cis*-eQTL mapping in the admixed population (117AX) to identify associations between variants and gene expression within each of the seven tissues indicated in Fig. 1b (see the “Methods” section; Additional file 5, Table S4). We implemented linear models to test for an association between each gene-*cis*-variant pair. For each pair, two association tests were performed: the first to adjust for global ancestry (GlobalAA) and the second to adjust for local ancestry (LocalAA). Importantly, LocalAA accounts for the number of European, African, and East Asian alleles for each variant while GlobalAA uses the first five genotype principal components as a proxy for global ancestry, implementing the same ancestry adjustment used in the GTEx eQTL calling pipeline.

**Fig. 2:**
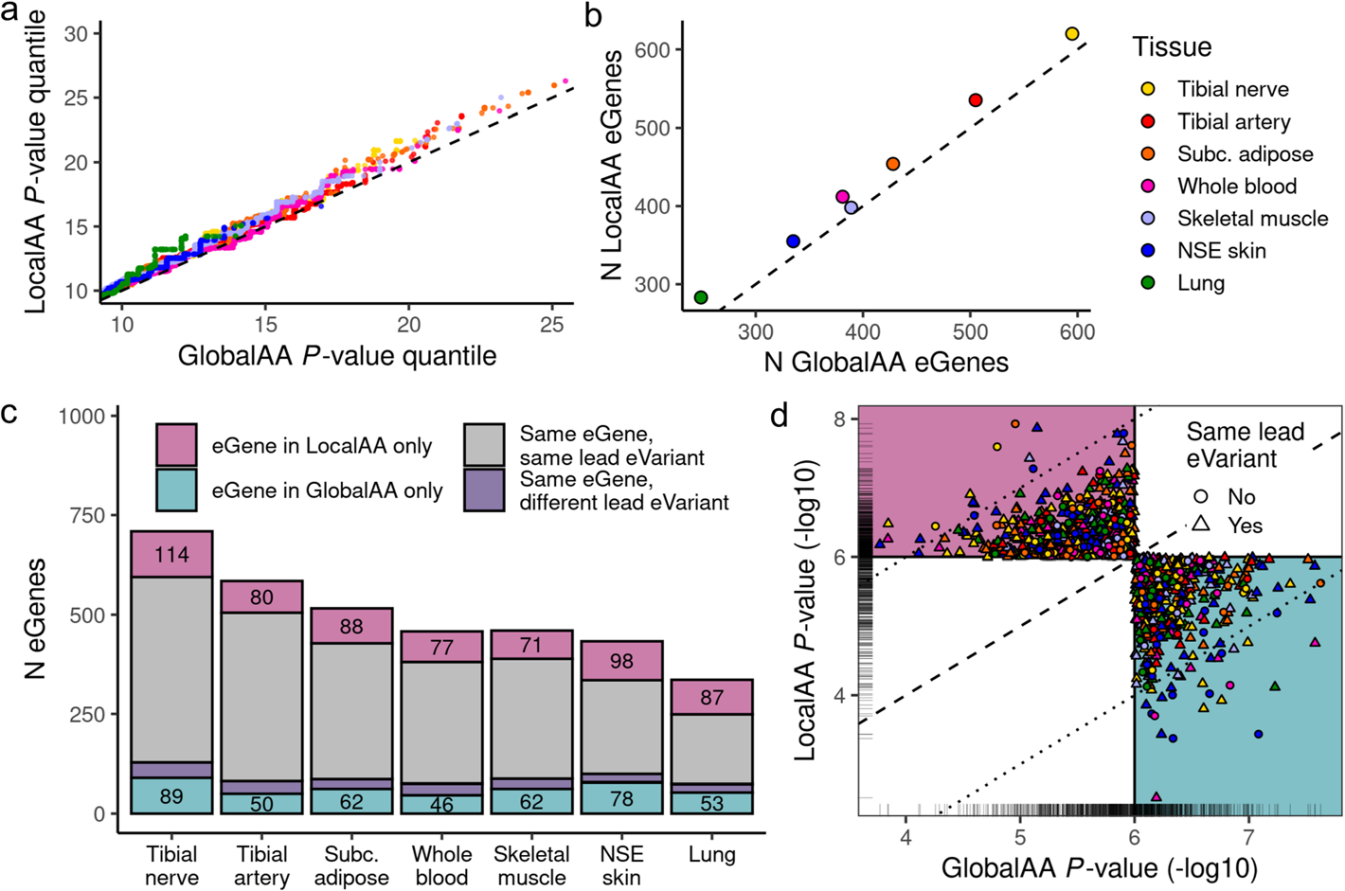
Comparison of *cis*-eQTLs called by LocalAA or GlobalAA. *Cis*-eQTL mapping was performed in seven tissues. A nominal *P* value threshold of 1e−6 was applied to identify significant associations. **a** A Q-Q plot of nominal *P* values for all tests indicates a modest improvement of power in most tissues when using LocalAA. **b** LocalAA identifies more eGenes than GlobalAA in all seven tissues (*P* value = 0.0078, binomial probability). **c** The majority of eGenes are identified by both ancestry adjustment methods (gray + purple). The two methods report different eVariants for a small fraction of these eGenes (purple). Numbers indicate eGenes uniquely called by one of the ancestry adjustment methods, which are plotted in **d**. **d** The majority of eGenes unique to one ancestry adjustment method fall near the significance threshold, as indicated by the rug plot. Dotted lines demarcate the region outside of which eGenes in one method have a nominal *P* value at least two orders of magnitude more significant than the alternate method. Points are colored by tissue

A quantile-quantile plot of the nominal *P* values (-log10) of all association tests in GlobalAA and LocalAA demonstrates that LocalAA has more significant *P* values (represented in the highest quantiles) relative to GlobalAA for six of the seven tissues, with NSE skin showing more similar *P* value distributions between the two methods (Fig. 2a). This corroborates previous findings that LA adjustment results in more significant nominal *P* values than GA adjustment in the context of *cis*-eQTL mapping [10].

We applied a nominal *P* value cutoff of 1e−6 to identify significant eQTLs; this threshold closely approximates the threshold required for an eQTL to subsequently pass a false discovery rate cutoff of 5% (Additional file 1, Figure S4). More eGenes are called with LocalAA than GlobalAA in all seven tissues (*P* value = 0.0078, binomial probability) (Fig. 2b). The majority of the eGenes overlap between the two methods, a subset of which has different associated lead eVariants between LocalAA and GlobalAA (Fig. 2c). This subset of eGenes provided an opportunity to characterize differences in lead eVariants identified between the two ancestry adjustment methods and was the focus of downstream analyses.

eGenes are considered unique to an ancestry adjustment method if the association reaches significance only with that method (nominal *P* value cutoff of 1e−6; 1055 total instances across tissues for 988 unique genes). The majority (65%) of eGenes that are unique to one method replicate at a *P* value within one order of magnitude of the other method (Fig. 2d). However, 44 of these eGenes only replicate in the other method at a *P* value more than two orders of magnitude less significant (14 and 30 eGenes unique to LocalAA and GlobalAA, respectively). Twenty of these 44 eGenes are in NSE skin; none is in the tibial artery. Interestingly, for 29 out of these 44 eGenes, despite the large difference in the statistical significance, the lead variants between the two adjustment methods are identical.

### Different eQTL ancestry adjustments yield minor differences in GWAS colocalization

Colocalization analyses assess the degree to which independent signals of association, including eQTL and GWAS signals, share the same causal variant. We performed colocalization with two different methods: COLOC [30] and FINEMAP [31]. COLOC estimates the posterior probability that a single variant affects both traits (PP4). FINEMAP estimates the posterior probability of single trait causality for all variants in a region; as previously described, these probabilities can be used to derive a colocalization posterior probability (CLPP) for two independent association signals [32] (see the “Methods” section). Importantly, FINEMAP explicitly accounts for linkage disequilibrium (LD) while COLOC does not; this is particularly relevant given the admixed ancestry of the eQTL cohort.

We selected 142 GWAS to perform colocalization with our eQTLs. Previously, 114 of these GWAS were used to perform colocalization with all GTEx v8 eQTLs [33]. These GWAS were originally chosen to include a broad representation of different trait classes and some replication between GWAS from the UK Biobank (UKB) and other consortia. We included an additional 28 multi-ethnic GWAS from the PAGE study to increase the representation of admixed cohorts in our colocalization analyses [34]. More information about each GWAS is available in Table S5 (Additional file 6).

**Fig. 3:**
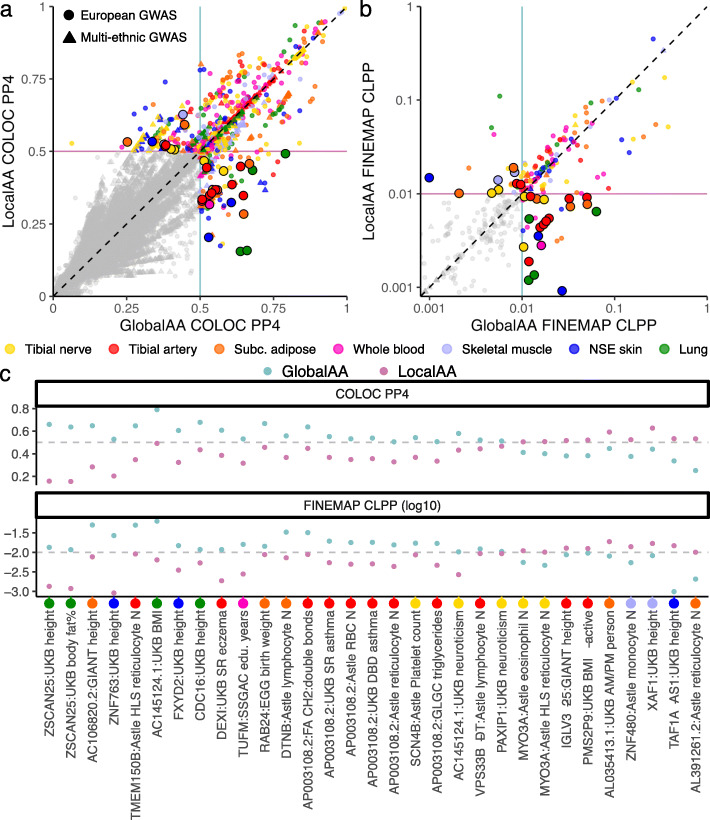
Impact of eQTL ancestry adjustment methods on colocalization with GWAS. **a**, **b** We performed colocalization for a subset of loci where LocalAA and GlobalAA called eQTLs with different lead eVariants (nominal *P* value threshold of 1e−4). Each point represents a GWAS/eQTL colocalization test near a single eGene (colored by eQTL tissue). The *x*- and *y*-axes respectively show the posterior probabilities of colocalization using either GlobalAA or LocalAA eQTL signals. The same 31 points highlighted in both plots correspond to loci where one ancestry-adjusted eQTL signal colocalized but the other did not, with concordant results between two colocalization methods. **a** Colocalization was performed with COLOC for all loci where LocalAA and GlobalAA called eQTLs with different lead eVariants (nominal *P* value threshold of 1e−4). A posterior probability of colocalization (PP4) threshold of 0.5 was used to identify colocalization events with COLOC. **b** For the subset of loci for which COLOC reported a colocalization (i.e., colored points in **a**), colocalization was also performed with FINEMAP. Colocalization posterior probabilities (CLPPs) are shown on a log10 scale. A CLPP threshold of 0.01 was used to identify colocalization events with FINEMAP. **c** Colocalization posterior probabilities are provided for the 31 loci highlighted in **a** and **b**. Larger values indicate stronger colocalization. The associated eQTL tissues are indicated with colored circles and tick marks below the *x*-axis. SR, self-reported; DBD, diagnosed by doctor; *N*, count

We performed colocalization between our fourteen sets of eQTL summary statistics (one per ancestry adjustment method per seven tissues) and 142 GWAS. Here, we define a locus as a gene and GWAS trait pair in a specific tissue. For a single locus, two colocalization tests are performed with each colocalization method: one test between the GWAS and each set of eQTL summary statistics (LocalAA or GlobalAA). Therefore, there are up to four colocalization scores (COLOC PP4 or FINEMAP CLPP) for a single locus. For colocalization analyses with COLOC, we restricted tested loci to the subset of eGenes with different lead eVariants between LocalAA and GlobalAA at a relaxed nominal *P* value threshold (Fig. 3a). We subsequently performed colocalization analyses with FINEMAP for the subset of loci with at least one COLOC colocalization (Fig. 3b). We define evidence for colocalization at a locus as PP4 > 0.5 or CLPP > 0.01 for COLOC and FINEMAP, respectively.

While GWAS colocalization was only tested at loci for which the two eQTL ancestry adjustment methods yielded different lead eVariants, colocalization probabilities are not systematically different between the two methods (*P* value = 0.791 and *P* value = 0.324 for COLOC and FINEMAP, respectively; two-sided *t* test). Furthermore, loci with strong evidence of colocalization (COLOC PP4 > 0.5 or FINEMAP CLPP > 0.01) have similarly high posterior probabilities of colocalization regardless of the correction method, indicating that robust effects are captured by both ancestry adjustments.

Of 174,388 loci tested for colocalization, 793 loci (< 0.5%) have at least one colocalization reported by *either* COLOC or FINEMAP. Only 159 of these loci have at least one concordant colocalization reported by *both* COLOC and FINEMAP (i.e., both methods report a colocalization for LocalAA or GlobalAA or both). For a subset of 31 loci, one ancestry-adjusted eQTL signal colocalized but the other did not, with concordant results between the two colocalization methods. Twenty-two and 9 loci demonstrate stronger colocalization with GlobalAA and LocalAA, respectively (highlighted points, Fig. 3a, b; Fig. 3c; Additional file 1, Figure S5). Interestingly, all 31 loci correspond with GWAS in primarily European cohorts, regardless of whether colocalization is stronger with GlobalAA or LocalAA.

Six of the loci with stronger GlobalAA colocalizations are associated with the same eGene, *AP003108.2* in the tibial artery. The six colocalized GWAS are associated with three types of traits: asthma (UKB self-reported asthma; UBK diagnosed-by-doctor asthma); red blood cell counts (Astle et al. red blood cell count; Astle et al. reticulocyte count); and fatty acids (GLGC triglycerides; MAGNETIC CH_2_:double bond ratio in circulating fatty acids). Despite this replicated colocalization, neither the unannotated gene *AP003108.2* nor the GlobalAA lead eVariant, rs492751, has reported associations in the GWAS Catalog [35]. We further observed that rs492751 has highly variable allele frequencies between 1000 Genomes superpopulations (alternative allele frequencies of 0.02, 0, and 0.76 in European, East Asian, and African populations, respectively). This suggests that these stronger colocalizations with the GlobalAA tibial artery *AP003108.2* eQTL signal may in fact be driven by spurious associations confounded by local ancestry. Notably, a stronger colocalization with one eQTL ancestry adjustment is not synonymous with a more accurate eQTL signal; confounded associations can yield false discoveries.

Two loci with stronger LocalAA colocalizations correspond with *MYO3A* in the tibial nerve. The associated traits are eosinophil counts and high light scatter reticulocyte counts (Astle et al.). *MYO3A* associations with interleukin-6, cortisol secretion, and BMI-adjusted waist circumference have previously been reported [35]; in other studies, eosinophil counts and characteristics of red blood cells have been correlated with obesity or BMI [36, 37], and obesity is associated with an inflammatory response [38, 39]. Therefore, a true colocalization between the tibial nerve *MYO3A* eQTL and traits related to properties of immature red blood cells and white blood cells is plausible. This locus provides an example of where LocalAA may outperform GlobalAA in terms of capturing true eQTL signals. However, we acknowledge that the differences in colocalization probabilities are smaller when LocalAA has a stronger colocalization compared to when GlobalAA has a stronger colocalization. In general, LocalAA may reduce false associations more often than it discovers true associations not also identified with GlobalAA. Overall, we observe that neither LocalAA nor GlobalAA performs significantly better in the context of colocalization, regardless of GWAS ancestry or colocalization method.

### A subset of GTEx v8 eVariants is highly correlated with local ancestry

**Fig. 4:**
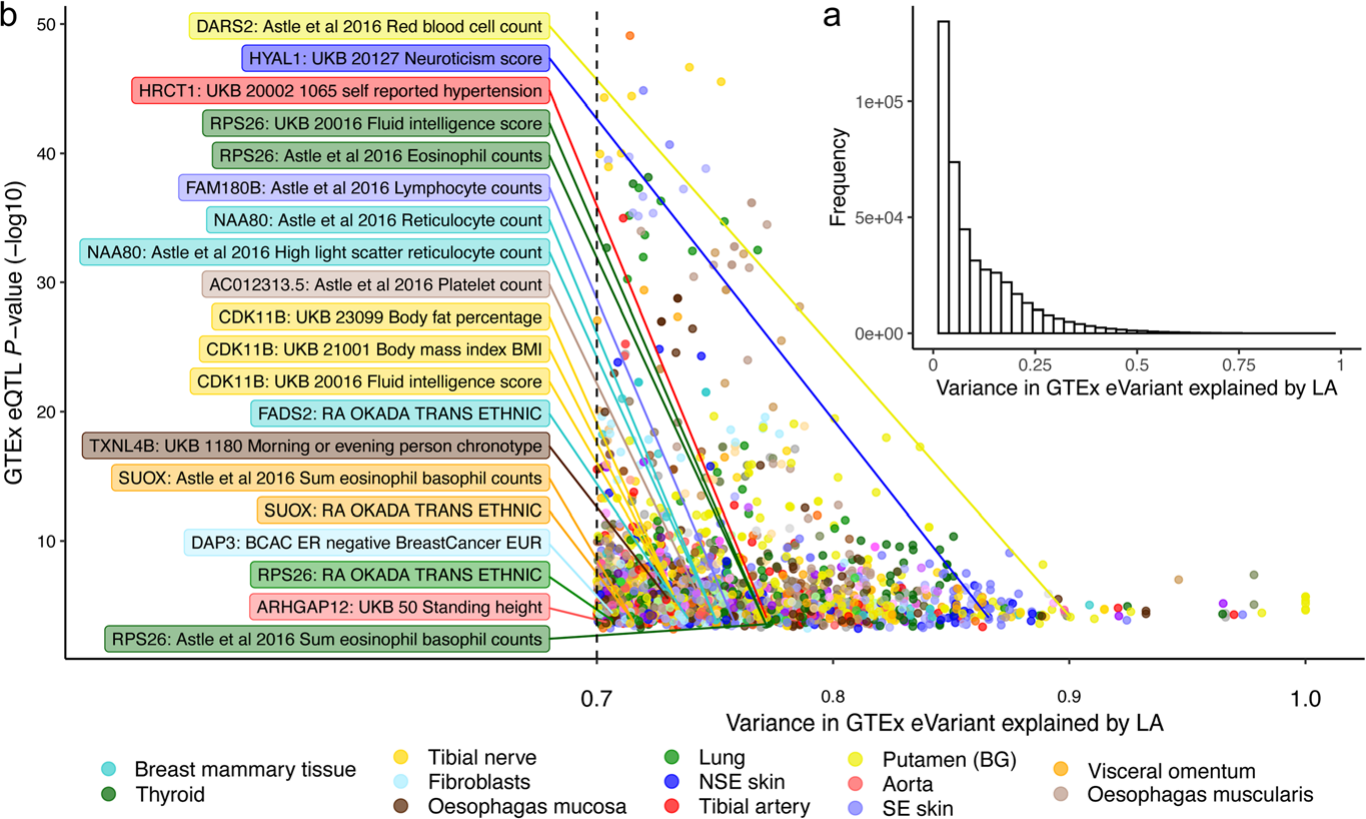
Correlation between genotype and local ancestry in GTEx v8 eVariants. For all eVariants reported by the overall GTEx v8 eQTL calling pipeline, we calculated the correlation between genotypes and local ancestry using the full GTEx v8 cohort. **a** The majority of GTEx v8 eVariants are not confounded by local ancestry when all 838 genotyped individuals are considered. **b** Local ancestry explains more than 70% of the variance in genotypes for a subset of GTEx v8 eVariants. Unlike **a**, **b** considers only individuals with matched genotype and gene expression data for each tissue, which reflects the sample used to call these significant associations. eQTLs with posterior probabilities of GWAS colocalization of at least 0.5 (COLOC PP4 > 0.5) are labeled with the eGene and GWAS trait

One justification for performing LocalAA as opposed to GlobalAA is the unique ability to avoid confounding by local population structure [15]. We examined all significant associations reported by the overall GTEx v8 eQTL calling pipeline for evidence of confounding with LA. Note that this analysis is expanded to include the full GTEx v8 cohort, not just the admixed sub-cohort involved in preceding analyses. For each GTEx eVariant in the set of all significant associations across 49 tissues, we found the variance in genotype explained by LA (the number of African and East Asian alleles at the locus) across all 838 genotyped individuals (see the “Methods” section). The vast majority of GTEx eVariants are not strongly correlated with LA when the entire genotyped population of 838 individuals is considered (Fig. 4a).

However, transcriptome sample sizes within each GTEx v8 eQTL tissue are often less than the full sample size (mean 310; standard deviation 171). Therefore, the degree of confounding between a variant’s genotype and LA in the context of eQTL mapping can vary between tissues. To this point, Fig. 4b provides the variance in genotype explained by LA for GTEx eVariants when only subjects with matched genotype and expression data are included in the regression. Unlike Fig. 4a, an eVariant has as many data points as tissues in which it is reported in a significant association. Twenty GTEx v8 eVariants whose corresponding eGenes have a colocalization probability of greater than 0.5, as reported by Barbeira et al., are also annotated [33]. Notably, 19 unique eVariants have proportions of variance explained by LA greater than 0.9 (Additional file 7, Table S6). These variants have large differences in reference allele frequencies between 1000 Genomes populations. For example, one such variant, chr1_1170732_A_G_b38, has reference allele frequencies of 0.993, 0.996, and 0.124 in European, East Asian, and African populations, respectively. A comprehensive list of the 2556 GTEx v8 significant associations where LA explains more than 70% of the variance in the eVariant genotype is provided in Table S7 (Additional file 8). We expect that functional follow-ups of eQTL/GWAS colocalizations will benefit from cross-referencing with these data.

## Discussion

In this study, we describe population admixture in the GTEx v8 release and assess the impact of ancestry adjustment on eQTLs discovered in an admixed sub-cohort (117AX).

GTEx expands representation from non-European populations, including up to 17% of non-European or admixed individuals. For eQTL mapping, the selection of tissues was limited to those with adequate 117AX sample sizes (> 60). We recognize that these relatively small sample sizes will remain an important limitation of multi-population analyses in the GTEx study. Future comparable multi-tissue studies will benefit from increased representation of diverse populations.

The observed trend that GA explains more variance in residual gene expression than LA, on average, agrees with the previous finding that GA explains significantly more heritability of gene expression than LA [40]. However, LA can explain a large proportion of variance in GA-corrected gene expression for a subset of genes. Interestingly, a gene whose expression is largely explained by LA, *TBC1D3*, is a highly expanded gene whose copy number is stratified by ancestral population [29, 41]. Given that copy number expansion is a local phenomenon that has limited effects on global gene expression, population differences in gene copy numbers creates a scenario in which we would expect LA to explain more variance in gene expression than GA. This biological explanation for the differences in *TBC1D3* expression explained by ancestry highlights a specific benefit of considering LA during eQTL mapping.

We decided to include only admixed samples in eQTL mapping on the basis that we would not expect LocalAA to perform any better than GlobalAA in homogeneously European individuals, where the LA covariates are expected to be constant across the majority of the genome. For this same reason, we also excluded homogeneously African (*N* = 14) and East Asian (*N* = 9) samples from eQTL calling. However, this does not preclude the use of LocalAA as an ancestry adjustment approach in a cohort with individuals of both homogeneous and heterogeneous ancestry. To this point, Zhong et al. reached similar conclusions when comparing LA and GA adjustments in either a strictly African American population or a cohort of mostly European-ancestry individuals with less than 25% African Americans [10].

After performing *cis*-eQTL mapping in seven tissues, we observe that LocalAA has a modest improvement in power, consistent with previous observations [10, 42]. We also observe that most eQTLs agree between LocalAA and GlobalAA; the majority of eGenes that are called uniquely by one ancestry adjustment method are at the threshold of significance. Both of these observations are consistent with previous findings by Zhong et al. [10]. Further, eGenes called uniquely by GlobalAA are not confounded by LA. Neither do differences in variance in gene expression explained by LA or GA explain these eGenes uniquely called by one method. This, combined with the fact that both methods indicate the same lead eVariant more often than not, even when the association only reaches significance with one method, suggests that eGenes uniquely called by GlobalAA may not in fact be driven by confounding with LA. Instead, LocalAA and GlobalAA may have relatively more power for eQTL discovery in different contexts.

To our knowledge, the effects of LA adjustment in eQTL mapping on GWAS colocalization have not previously been explored. In general, stronger colocalization events are captured by both ancestry adjustment methods. For 31 loci, only one of the two ancestry-adjusted eQTLs colocalizes with the GWAS, reported by both COLOC and FINEMAP. Interestingly, all 31 loci correspond with GWAS with European cohorts; no loci from the multi-ethnic GWAS robustly colocalize more strongly with either LocalAA or GlobalAA eQTLs. Six loci with stronger GlobalAA colocalization correspond with *AP003108.2* in the tibial artery; the GlobalAA lead eVariant has large differences in superpopulation allele frequencies, suggesting that confounding with local population structure is driving a spurious association signal. We also describe stronger colocalizations with LocalAA *MYO3A* eQTL signals in the tibial nerve that are supported by previously reported phenotypic associations. However, we find that neither LocalAA nor GlobalAA in eQTL mapping of seven different tissues yield systematically stronger colocalizations across 142 GWAS. Limitations of our colocalization analyses include our use of the assumption of one causal variant per trait and our lack of an attempt to colocalize secondary signals [43, 44].

Population-stratified eQTL calling is another potential approach in heterogeneous cohorts. To our knowledge, population-stratified eQTL calling has not yet been performed in the GTEx v8 cohort. However, the consortium did characterize population-biased *cis*-eQTLs (pb-eQTLs), where a variant’s molecular effect on gene expression differs between individuals of European and African ancestry [1]. Only 178 pb-eQTLs for 141 unique eGenes (FDR ≤ 25%) were identified across 31 tissues, which indicates that pb-eQTLs are hard to find and generally have small effects. Relatedly, Mogil et al. performed population-stratified eQTL calling independently in African American, Hispanic American, and European American samples in MESA; among several replication cohorts, the highest replication rate for all three discovery populations was in the Framingham Heart Study, a European cohort, simply because the sample size was much larger than the other population-matched replication cohorts [45]. This result, combined with the paucity of eQTLs with robust differences in effect sizes between populations, suggests that population-stratified eQTL calling at current sample sizes is limited in its ability to discover eQTLs not found in a pooled analysis.

One limitation of local ancestry inference is its dependence on the availability of appropriate reference panels. Access to genetic data for some populations remains limited, which makes it challenging to estimate local ancestry from those groups [26, 46]. Even with access to sufficient numbers of reference panels, there is a limit to the resolution that can be achieved with local ancestry inference given that local ancestry becomes more difficult to estimate as the genetic similarity between reference populations increases [11]. Addressing these challenges in future, larger functional genomics studies stands to improve our understanding of genetic risk across populations [47, 48] and resolution for the identification of causal variants [49].

Finally, the additional step of LA inference and the incorporation of LA into models for eQTL calling or GWAS makes LocalAA much more computationally intensive than GlobalAA. Therefore, a significant improvement of power for discovery or fine-mapping would be required to motivate the widespread implementation of LocalAA in large genetic association studies. Several groups recommend that GlobalAA is sufficient to control for type I error during screening for genetic associations, but LocalAA at loci of interest may improve fine-mapping or provide better effect estimates [5, 8, 9, 18]. Thus, a candidate approach may be taken to adjust for LA only at a subset of loci where LA is expected to improve fine-mapping, which would reduce computational cost and maximize the potential benefit of LA adjustment.

A practical example of this is performing eQTL mapping with GlobalAA and subsequently assessing residual variance explained by LA for discovered eQTLs. To assess this, we post hoc analyzed GTEx release eVariants to discover 2556 associations that have a large amount of variance explained by local ancestry (> 70%). It remains a challenge to select a threshold for simply excluding QTLs based on the degree of variance explained by local ancestry. We provide this list to enhance the future analysis of eQTL/GWAS associations.

## Conclusions

Despite claims of the importance of accounting for LA when performing genetic association studies in admixed populations [15, 16], the impact of LocalAA in the context of eQTL mapping and GWAS interpretation has been relatively underexplored. We performed genome-wide LA inference in an admixed sub-cohort of GTEx v8 and provide these LA calls as a resource to further investigate GTEx data. We then performed *cis-*eQTL mapping in this admixed sub-cohort to compare GlobalAA and LocalAA ancestry adjustment methods. We observe a modest improvement in power with LocalAA relative to GlobalAA. While both methods yield the same lead eVariant for the majority of eGenes, small subsets of eGenes have different lead eVariants between methods or pass the eQTL significance threshold in only one of the methods. We do not see large-scale or systematic differences in colocalization probabilities when we perform colocalization between GWAS and eQTLs where the two ancestry adjustments yield different lead eVariants. Finally, we provide a resource of GTEx v8 eVariants that are potentially confounded by LA. Together, these results describe the population structure of admixed individuals in the GTEx v8 release and demonstrate limited confounding based on local ancestry.

## Methods

### Genotype data

We used GTEx v8 release genotype data [1]. Briefly, whole genome sequencing (WGS) was performed for 899 samples from 869 unique GTEx donors, to a median depth of 32×. Alignment to the human reference genome build GRCh38 was performed with BWA-MEM [50]. Variants were called with GATK HaplotypeCaller v3.5, and multi-allelic sites were split into biallelic sites using Hail v0.1 [51]. After performing quality control, the final analysis freeze set contained variant calls from 838 donors. SHAPEIT v2 [52] was used to impute missing calls and phase the sample- and variant-QCed variant call file (VCF).

### Genotype principal component analysis

We used GTEx v8 release genotype principal components (gPCs) [1]. gPCs were computed based on the sample- and variant-QCed WGS VCF using EIGENSTRAT [6]. PCA was performed on a set of LD-independent variants with a call rate ≥ 99% and MAF ≥ 0.05. LD pruning was performed using PLINK 1.9 [53].

### Gene expression data

We used GTEx v8 release normalized gene expression data; detailed method descriptions can be found in the main GTEx publication [1]. RNA sequencing (RNA-seq) was performed at the Broad Institute using the Illumina TruSeq™ RNA sample preparation protocol, which was based on polyA+ selection of mRNA and was not strand-specific. RNA-seq data were aligned to the human reference genome GRCh38/hg38 with STAR v2.5.3a [54]. Gene-level expression quantification was performed using RNA-SeQC [55] with a gene annotation available on the GTEx Portal (gencode.v26.GRCh38.genes.gtf). Quantified gene expression (TPM and raw counts) for each tissue was filtered and normalized according to the GTEx eQTL discovery pipeline [56]. For each of the seven tissues in which we chose to perform eQTL mapping, we subsetted normalized gene expression to include only 117AX samples.

### Local ancestry inference

LiftOver [57] was used to convert the phased GTEx v8 whole genome sequencing variant call file (VCF) (dbGaP accession number phs000424.v8) from reference genome Human Build 38 (hg38) to Human Build 37 (hg19) for compatibility with 1000 Genomes and the hg19 HapMap genetic map. The resulting GTEx VCF was filtered to include self-reported African Americans and Asian Americans (103 and 12 individuals, respectively) as well as 25 admixed individuals as identified by the genotype PCA (Fig. 1a), resulting in 140 individuals. 1000 Genomes Phase 3 phased VCFs [58] were filtered to include biallelic variants and only individuals in the following populations: Han Chinese in Beijing, China (CHB); Japanese in Tokyo, Japan (JPT); Utah residents (CEPH) with Northern and Western European ancestry (CEU); Yoruba in Ibadan, Nigeria (YRI); Gambian in Western Divisions in the Gambia (GWD); Mende in Sierra Leone (MSL); and Esan in Nigeria (ESN) [19]. The intersection of autosomal variants in the resulting GTEx and 1000 Genomes VCFs (*N* = ~ 28 M) was identified for LA inference. For compatibility with RFMix v1.5.4, variant positions were converted from base pairs to centimorgans [59] using the HapMap hg19 genetic map [60].

RFMix v1.5.4 [61] was run in PopPhased mode with the additional --forward-backward option [26]. All other parameters were set to the default values. The 1000 Genomes populations were used as reference panels for European (EUR), East Asian (ASN), and African (AFR) populations as follows: EUR (CEU, *N* = 99), ASN (CHB, JPT, *N* = 207), and AFR (YRI, GWD, MSL, ESN, *N* = 405) [19]. This generated posterior probabilities for the assignment of each phased allele to each of the three reference populations (EUR, AFR, ASN). An allele was assigned to a reference population only if the posterior probability was at least 0.9; otherwise, the local ancestry was indicated as “unknown.” For each individual, consecutive phased alleles with the same LA assignment were collapsed into BED files of haplotype blocks with the same LA (Additional file 3, Table S2). These BED files were then used to calculate global ancestry fractions per individual. Scripts used to collapse LA into BED files and calculate global ancestry fractions are available [62].

Of the 140 GTEx v8 individuals whose LA was inferred, 117 individuals with less than 90% global ancestry in a single population (among EUR, AFR, and ASN) were defined as admixed and retained for downstream analyses. This cohort is referred to as 117AX in this paper. VCFtools [63] was used to filter the hg19 GTEx VCF down to variants with a minor allele count (MAC) of at least 10 in 117AX. For the remaining 8,088,666 variants, the LA BED files (Additional file 3, Table S2) were used to count the number of EUR, AFR, ASN, and unknown alleles at each SNP within 117AX. These allele counts were used as LA covariates in eQTL mapping with LocalAA.

### Variance in gene expression explained by ancestry

We adapted an existing approach [27] to quantify variance in gene expression explained independently by LA or GA. For each expressed gene in each tissue, we performed two-step regressions to quantify variance explained by LA (or GA) in the gene expression residualized by GA (or LA). First, we regressed out the effects of one type of ancestry (LA or GA) on the gene expression using the following multiple linear regression, where *γ*_*i*_ is the effect of ancestry covariate *a*_*i*_ on gene expression *g*, and *e*_*g*_ is the residual:

$$ g=\sum \limits_{i=1}^m{\gamma}_i{a}_i+{e}_g $$

*m* is five for GA (five genotype PCs) and two for LA (numbers of alleles assigned to African or East Asian ancestry at the gene’s transcription start site). Then, we quantified variance in *e*_*g*_ explained by the other type of ancestry (*a*^∗^, LA or GA covariates) by taking the coefficient of determination from the following linear regression:

$$ {e}_g=\sum \limits_{i=1}^m{\gamma}_i{a}_i^{\ast }+\epsilon $$

This process was performed for both LA and GA. All regressions were performed with the lm() function in R.

### *cis*-eQTL mapping with LocalAA and GlobalAA

Genome-wide *cis*-eQTL mapping in 117AX was performed in seven GTEx v8 tissues: subcutaneous adipose (subc. adipose), tibial artery, lung, skeletal muscle, tibial nerve, whole blood, and not-sun-exposed suprapubic skin (NSE skin). All methods in this section were performed independently for each tissue. Normalized gene expression files filtered to include only 117AX samples were used to calculate 15 hidden confounders with PEER [64] according to the GTEx eQTL discovery pipeline [56]. Additional sample-level covariates, including gPCs, WGS sequencing platform (HiSeq 2000 or HiSeq X), WGS library construction protocol (PCR-based or PCR-free), and donor sex, were extracted from GTEx v8 release covariate files.

We assumed an additive genetic effect on gene expression and fit the following linear model for each gene-variant pair (gene *g*, variant *v*):

$$ G=\beta V+\sum \limits_{i=1}^k{\alpha}_i{c}_i+\sum \limits_{i=1}^m{\gamma}_i{a}_i+e $$

where *G* is the expression of gene *g* across 117AX samples in the given tissue; *V* is the number of alternate alleles at variant *v*, coded as 0, 1, or 2; *β* is the effect of the alternate allele of variant *v* on gene *g* expression; *α*_*i*_ is the effect of the technical or biological covariate *c*_*i*_ on gene *g* expression, including donor sex, sequencing platform, library construction protocol, and fifteen hidden confounders; *γ*_*i*_ is the effect of ancestry covariate *a*_*i*_ on gene *g* expression; and *e* is the residual. Any of the 8,088,666 filtered variants within a megabase of the transcription start site of a gene were tested for an association with that gene’s expression. The significance of an association was taken to be the two-sided *P* value corresponding to the *t*-statistic of the *β* coefficient estimate. All regressions were performed with the lm() function in R.

For each gene-variant pair, two iterations of this regression were performed: one to adjust for global ancestry (GlobalAA), in which case each *a*_*i*_ is one of the first five genotype principal components (gPCs), and one to correct for local ancestry (LocalAA), in which case there are two ancestry covariates, coded as the number of alleles at variant *v* assigned to African and East Asian populations, respectively. gPCs were not included as covariates in the LocalAA model. For LocalAA, samples with any number of alleles with unknown ancestry for the given variant were excluded; the covariate matrix was necessarily reconstructed for each variant tested. This is unlike GlobalAA, where the GA covariates are also sample-level covariates and can be reused for every association test.

After eQTL mapping was completed, the most significant, i.e., lead, eVariant (or eVariants, in the case of tied *P* values) was identified for each gene, independently for the two ancestry adjustment methods. A nominal *P* value cutoff of 1e−6 was applied to identify significant associations. This threshold approximates a 5% FDR (Additional file 1, Figure S4). LD (*R*^2^) was calculated between single pairs of GlobalAA and LocalAA lead eVariants for each eGene using PLINK [53]; an eGene was defined as having different lead eVariants between the two ancestry adjustment methods if (1) there was no intersection between the two sets of lead eVariants and (2) the LD between the tested pair of GlobalAA and LocalAA lead eVariants was less than 1.0.

### Variance in GTEx eVariant genotype explained by local ancestry

In order to identify potential confounding by LA in GTEx v8 eQTLs, we first needed LA calls for all 838 individuals with both WGS and RNA-seq data [1]. The remaining 698 individuals for which we did not perform LA inference have self-reported European ancestry and cluster tightly together in gPC space (Fig. 1a). Therefore, we approximated LA in these 698 individuals to two European alleles at all tested loci. Then, LA covariates for this analysis were the union of computationally inferred LA in 140 admixed or non-European individuals and approximated LA in the remaining 698 homogeneously European individuals.

We calculated the variance explained by LA in the genotype of each eVariant implicated in reported GTEx v8 eQTLs. The following linear model was fit for each eVariant:

$$ V=\alpha \times \mathrm{AFR}+\beta \times \mathrm{ASN}+e $$

where *V* is the genotype vector (number of minor alleles), and *AFR* and *ASN* are the two LA covariate vectors, representing the number of alleles assigned to African and East Asian populations, respectively. The resulting coefficient of determination of each regression was recorded. We did this in two settings: (1) for the set of unique eVariants across all GTEx v8 eQTLs, where genotypes and LA for all 838 individuals were included in the regression (Fig. 4a), and (2) for all eVariants within each tissue, with samples subset to those with matched gene expression in the given tissue (Fig. 4b). (1) provides a global picture of the degree of correlation between eVariant genotypes and LA while (2) reflects the actual samples used to call eQTLs in each tissue. For (2), we also intersected GTEx v8 eQTLs with GTEx v8 GWAS colocalization results (see below) to identify loci with high posterior probabilities of colocalization between eQTLs and GWAS (PP4 > 0.5) associated with eVariants whose genotypes are highly correlated with LA (*R*^2^ > 0.7).

### Imputation of GWAS summary statistics

Harmonization and imputation of 114 previously published GWAS are described in detail by [33] and [1]. Briefly, summary statistics were harmonized and lifted over to hg38; an in-house implementation of best linear unbiased prediction (BLUP) [65, 66] was used to impute *z*-scores for those variants reported in GTEx without matching data in the GWAS summary statistics.

### Colocalization between eQTL and GWAS signals

We performed colocalization between 142 GWAS and 14 sets of 117AX eQTL summary statistics (one set for each ancestry adjustment in each of seven tissues). Colocalization tests were restricted to the subset of genes where the two eQTL ancestry adjustments yielded different lead variants with nominal *P* values less than 1e−4. COLOC was used to test for colocalization at all of these loci [30]; an implementation of FINEMAP was used to test for colocalization at the subset of loci for which COLOC reported a colocalization (PP4 > 0.5) [31]. Inputs were prepared similarly for COLOC and FINEMAP analyses. Each GWAS was scanned for putative association signals, defined as variants with a nominal *P* value less than 1e−5. If multiple variants within a 1-MB window had a *P* value less than this threshold, the variant with the smallest *P* value was selected as the seed variant. For each GWAS seed variant, if there was an eQTL with a *P* value of less than 1e−4 within 1 MB, the intersection of GWAS and eQTL variants within 1 MB of the GWAS seed variant was tested for colocalization. The same GWAS seed variant was used to perform colocalization with GlobalAA and LocalAA eQTL signals at each locus. Colocalization method-specific parameters are detailed below.

#### COLOC

For colocalization analyses between 142 GWAS and 117AX eQTL summary statistics, the same GTEx VCF used for eQTL mapping in 117AX was used to calculate eQTL effect allele frequencies; GWAS effect allele frequencies were extracted from the GWAS summary statistics. The coloc.abf() function in the “coloc” R package was used to run COLOC. For binary GWAS traits, case proportion and “cc” trait type parameters were used. For continuous GWAS traits, sample size and “quant” trait type parameters were used. These GWAS characteristics are provided in Table S5 (Additional file 6).

Figure 4b references colocalizations identified by an independent analysis of the 114 imputed GWAS and eQTLs reported in the GTEx v8 release [33]. Briefly, COLOC was used to perform colocalization with variants in the *cis*-window of each gene with at least one eVariant (*cis*-eQTL per-tissue *q* value < 0.05). For binary GWAS traits, case proportion and “cc” trait type parameters were used. For continuous GWAS traits, sample size and “quant” trait type parameters were used. In both cases, imputed or calculated *z*-scores were used as effect coefficients in Bayes factor calculations. Enloc enrichment estimates [67] were used to define data-based priors for COLOC in a consistent manner with other GTEx companion papers [33].

#### FINEMAP

An implementation of FINEMAP was used to test for colocalization at the subset of loci for which COLOC reported a colocalization (PP4 > 0.5) between a GWAS and an 117AX eQTL. After the intersection of GWAS and eQTL variants within 1 MB of the GWAS seed variant was identified for a locus, FINEMAP v1.1 was run independently for the GWAS and eQTL association signals using parameters --n-causal-max 1 --n-iterations 1000000 --n-convergence 1000. The 1000 Genomes Phase 3 VCF was used for LD calculations [19]. As previously described [32], the marginal posterior inclusion probabilities (PIPs) for each of *K* variants were then multiplied to calculate a colocalization posterior probability (CLPP):

$$ \mathrm{CLPP}=1-\left[\prod \limits_{i=1}^K1-\left({\mathrm{PIP}}_{\mathrm{GWAS},i}\times {\mathrm{PIP}}_{\mathrm{eQTL},i}\right)\right] $$

PIP_GWAS, *i*_ is the PIP for the *i*th variant in the vector of *K* variants tested for the causality of the GWAS signal; PIP_eQTL, *i*_ is the PIP for the *i*th variant in the vector of *K* variants tested for the causality of the eQTL signal. The *i*th variant in the list of tested GWAS variants is the same as the *i*th variant in the list of tested eQTL variants for all *i*.

## Supplementary information

**Additional file 1: Figure S1.** Supplementary figures (Figures S1-S5).

**Additional file 2: Table S1.** GTEx IDs for individuals in 117AX cohort.

**Additional file 3: Table S2.** Local ancestry calls for 140 GTEx individuals.

**Additional file 4: Table S3.** Variance in residual 117AX gene expression explained by ancestry.

**Additional file 5: Table S4.** 117AX GlobalAA and LocalAA lead eVariants for all tested genes.

**Additional file 6: Table S5.** Characteristics of GWAS used in colocalization analyses.

**Additional file 7: Table S6.** GTEx v8 eVariants highly correlated with LA (R^2^ > 0.9).

**Additional file 8: Table S7.** GTEx v8 eVariants correlated with LA (R^2^ > 0.7).

**Additional file 9.** GTEx Consortium author list.

**Additional file 10.** Review History.

## Data Availability

GTEx v8 release gene expression data and *cis*-eQTL call sets are available through the GTEx Portal [68]. GTEx v8 genotype data are available through the dbGaP website under dbGaP accession phs000424.v8.p2 [69]. LocalAA and GlobalAA eQTL summary statistics and colocalization posterior probabilities are available through Zenodo [70]. The 114 GWAS summary statistics imputed and harmonized by the GWAS GTEx Subgroup are available through Zenodo [71]. The source code is published on Zenodo under a Creative Commons Attribution 4.0 International License and available on GitHub [72]; a publication version of the code has been deposited on Zenodo [73].
