# Supplementary material for: Impact of admixture and ancestry on eQTL analysis and GWAS colocalization in GTEx
Source: Genome Biol. 2020 Sep 11;21:233. doi: 10.1186/s13059-020-02113-0 (PMC7488497; doi:10.1186/s13059-020-02113-0)
Supplement: Supplementary file 1 — Additional file 1: Figure S1. Supplementary figures (Figures S1-S5). [file 13059_2020_2113_MOESM1_ESM.pdf]

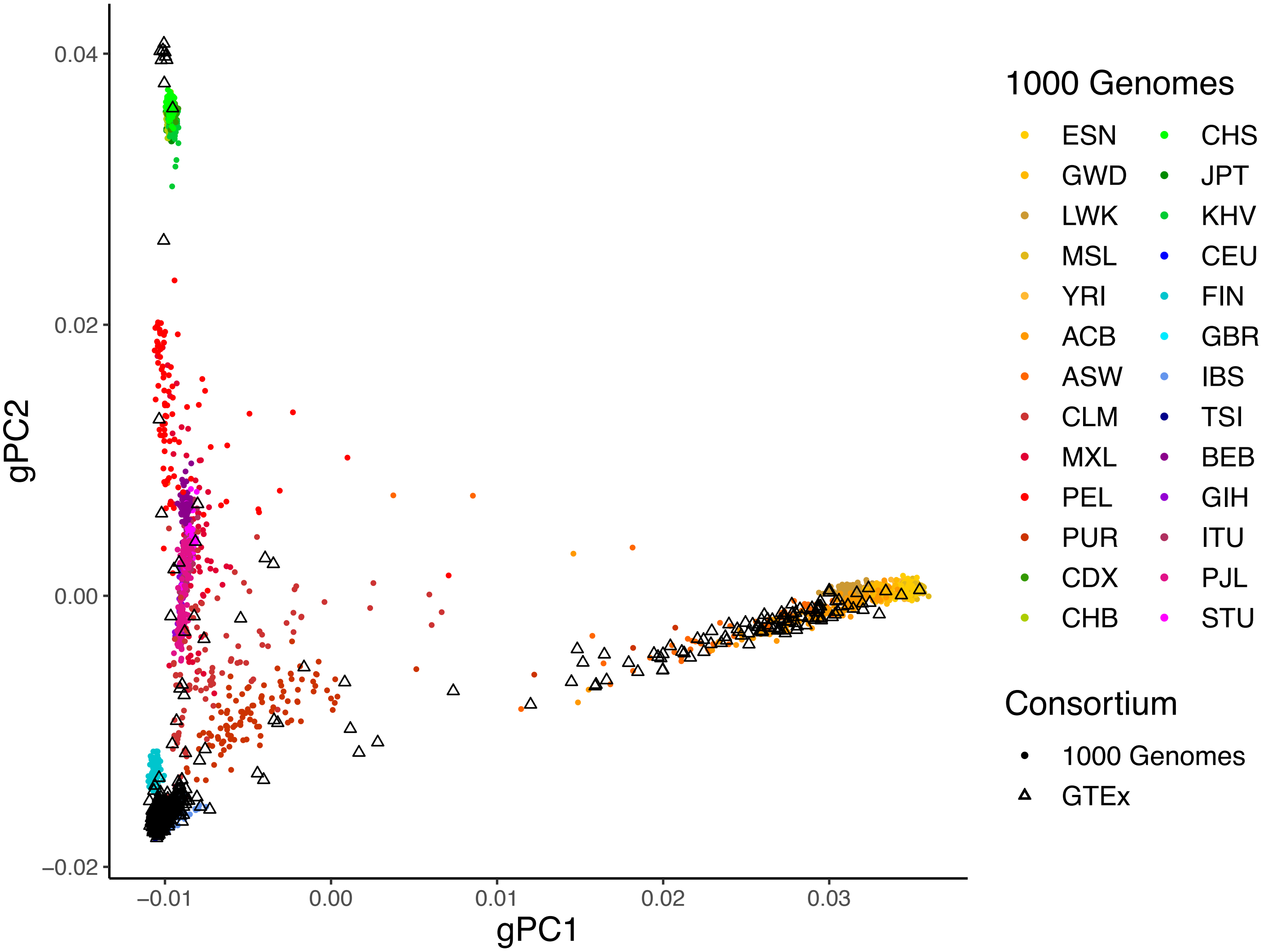

Figure S1. Spatial distribution of GTEx v8 subjects among 1000 Genomes populations. Genotype principal component analysis (gPCA) was performed with combined GTEx v8 and 1000 Genomes genotype data. As in Figure 1a;d, gPC1 is correlated with African ancestry; gPC2 is correlated with Asian ancestry. gPCA was performed using the *snpgdsVCF2GDS()* and *snpgdsPCA()* functions in the SNPRelate R package.

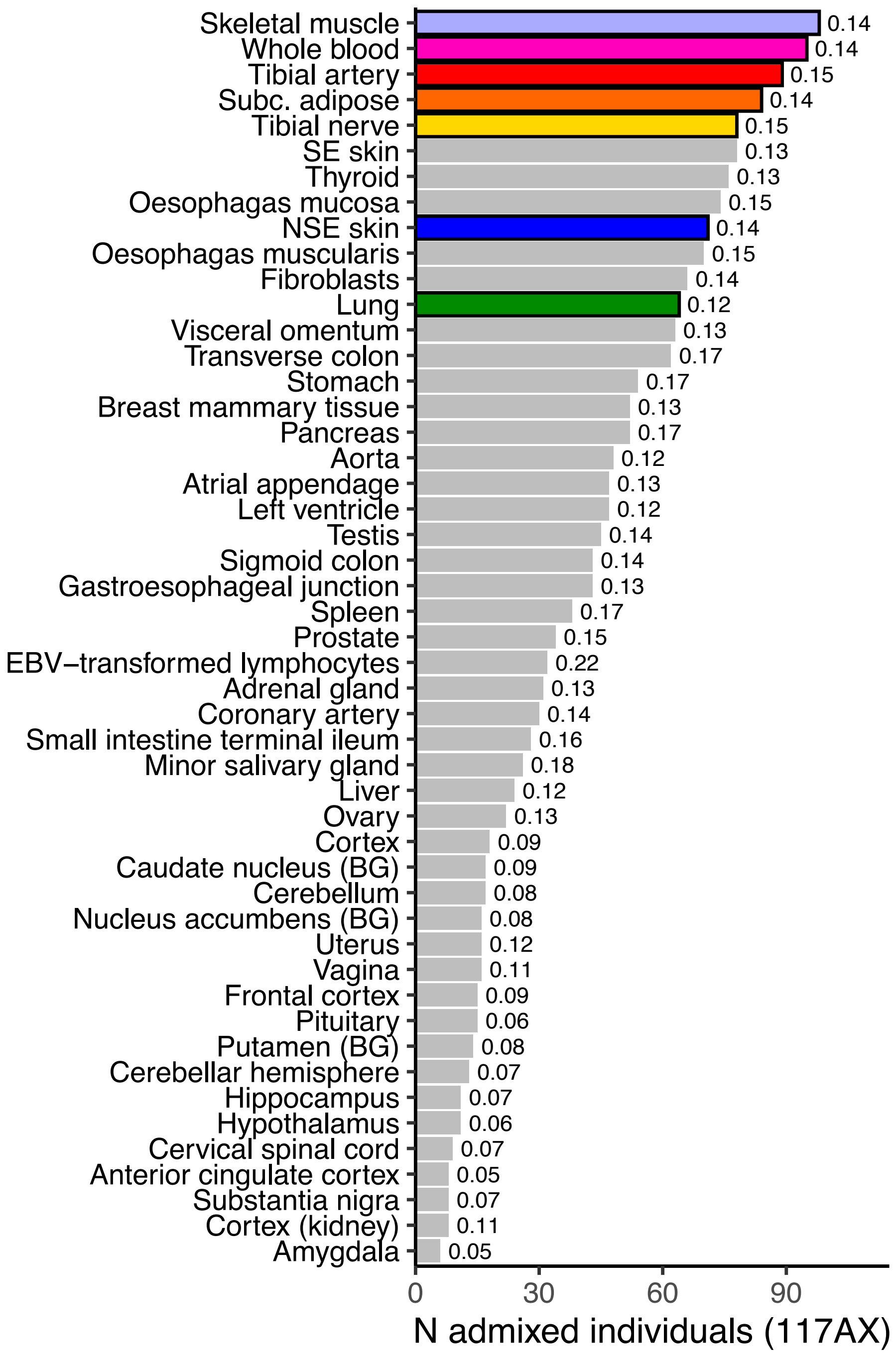

Figure S2. 117AX sample sizes vary across GTEx v8 tissues.
The fraction of samples within each tissue that correspond to 117AX samples are indicated to the right of each bar. 117AX represent 0.14 of the total 838 genotyped individuals in GTEx v8. The seven tissues selected for eQTL mapping in 117AX are colored.

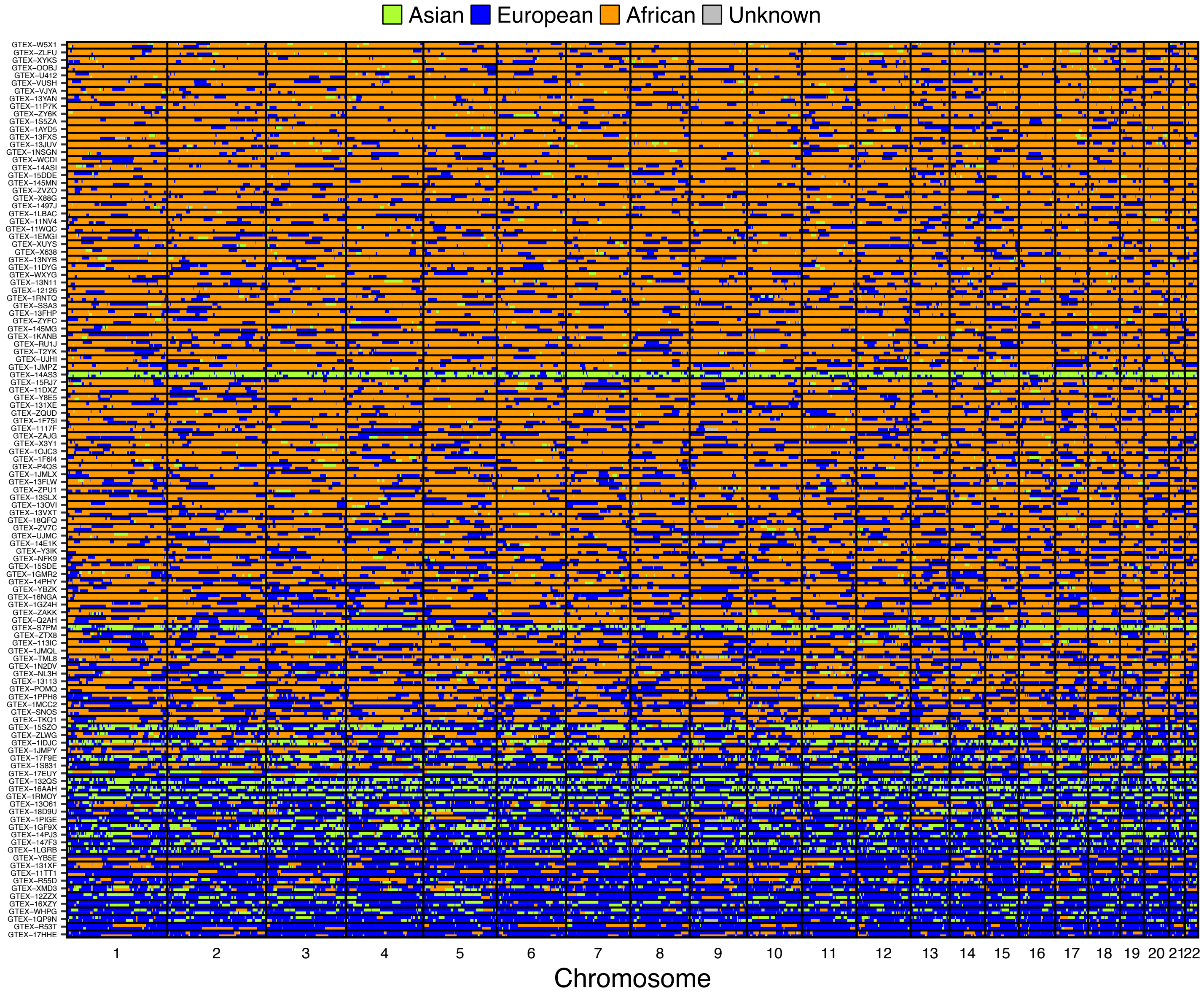

Figure S3. Genome-wide local ancestry in 117AX.
Each row provides a visual representation of the local ancestry calls in a haplotype of one individual across autosomal chromosomes (rows). Haplotypes are paired by individual, labelled by GTEx subject ID. Individuals are ordered from top to bottom with increasing amounts of European admixture.

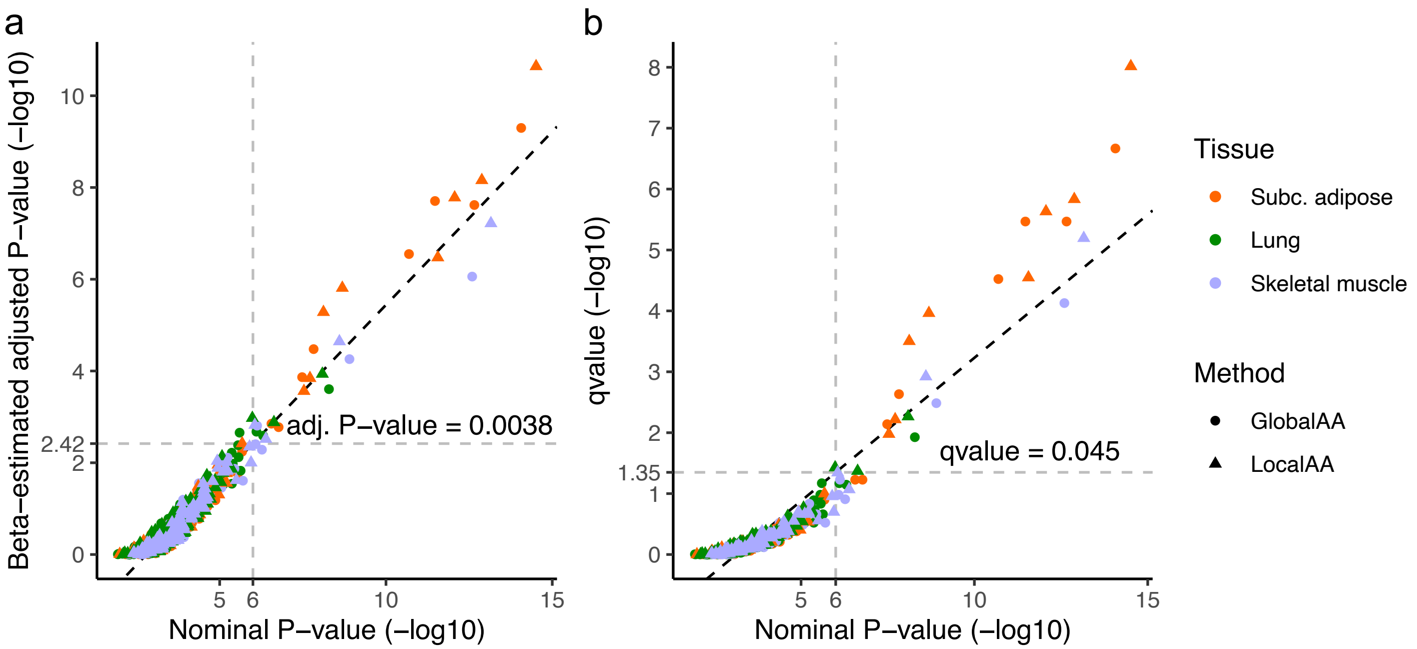

Figure S4. Approximation of FDR.

Permutation-based correction for multiple testing is computationally intensive for eQTL analysis with variant-specific covariates. To demonstrate that a nominal *P*-value of 1e-06 roughly corresponds to a genome-wide false discovery rate (FDR) of 5%, we performed gene-level Beta approximation followed by Storey's qvalue estimation for a subset of genes on chromosome 22 in three tissues. **(a)** A nominal *P*-value of 1e-06, indicated by the vertical dashed line, corresponds to a gene-level Beta-approximated adjusted *P*-value of 0.0038, as calculated by linear regression. **(b)** Qvalues (false discovery rates) were estimated from the vector of beta-approximated *P*-values. A nominal *P*-value of 1e-06, indicated by the vertical dashed line, corresponds to a qvalue of 0.045 for these loci, as calculated by linear regression.

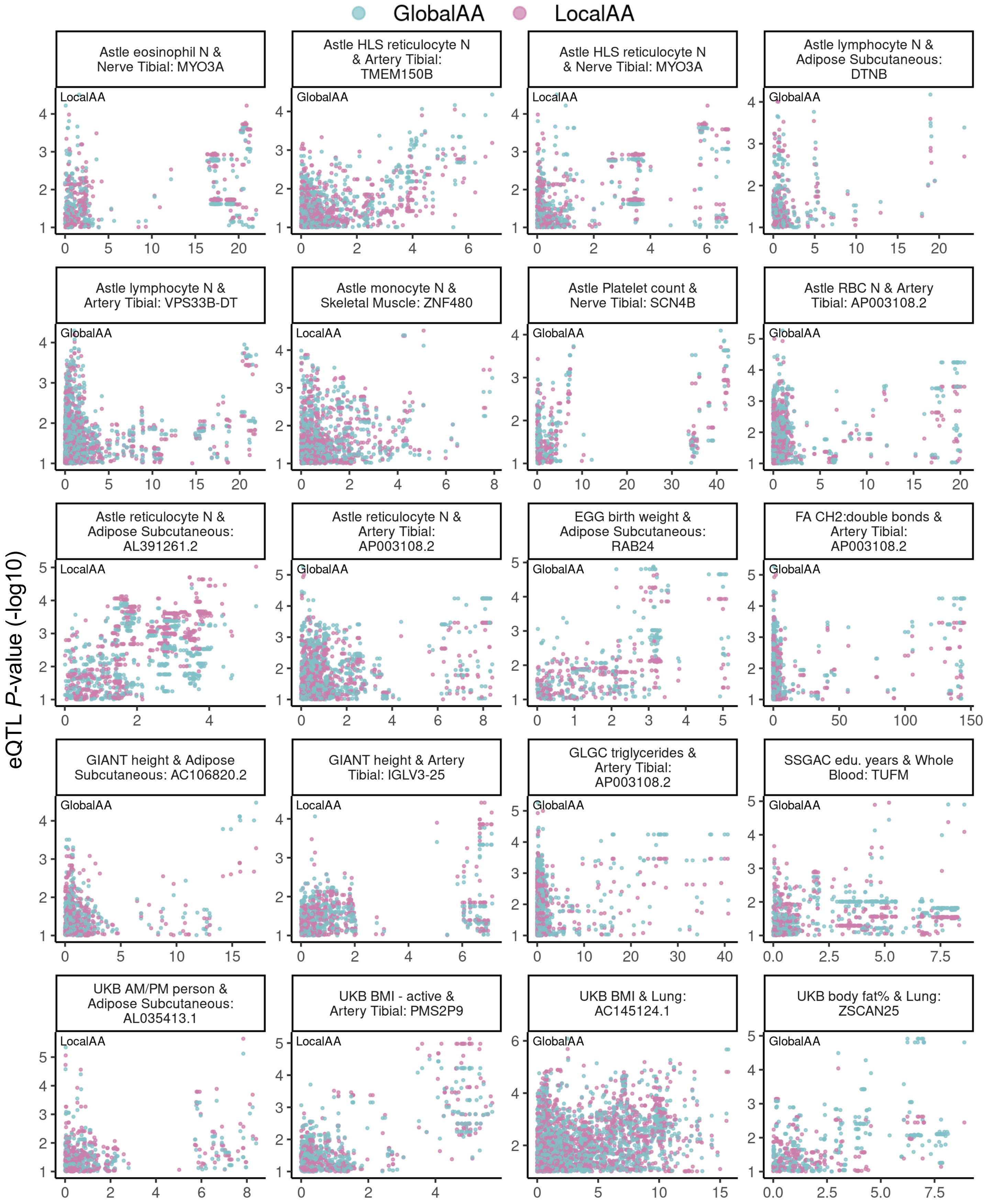
GlobalAA
LocalAA
eQTL P-value (-log10)
Astle eosinophil N & Nerve Tibial: MYO3A
Astle HLS reticulocyte N & Artery Tibial: TMEM150B
Astle HLS reticulocyte N & Nerve Tibial: MYO3A
Astle lymphocyte N & Adipose Subcutaneous: DTNB
Astle lymphocyte N & Artery Tibial: VPS33B-DT
Astle monocyte N & Skeletal Muscle: ZNF480
Astle Platelet count & Nerve Tibial: SCN4B
Astle RBC N & Artery Tibial: AP003108.2
Astle reticulocyte N & Adipose Subcutaneous: AL391261.2
Astle reticulocyte N & Artery Tibial: AP003108.2
EGG birth weight & Adipose Subcutaneous: RAB24
FA CH2:double bonds & Artery Tibial: AP003108.2
GIANT height & Adipose Subcutaneous: AC106820.2
GIANT height & Artery Tibial: IGLV3-25
GLGC triglycerides & Artery Tibial: AP003108.2
SSGAC edu. years & Whole Blood: TUFM
UKB AM/PM person & Adipose Subcutaneous: AL035413.1
UKB BMI - active & Artery Tibial: PMS2P9
UKB BMI & Lung: AC145124.1
UKB body fat% & Lung: ZSCAN25

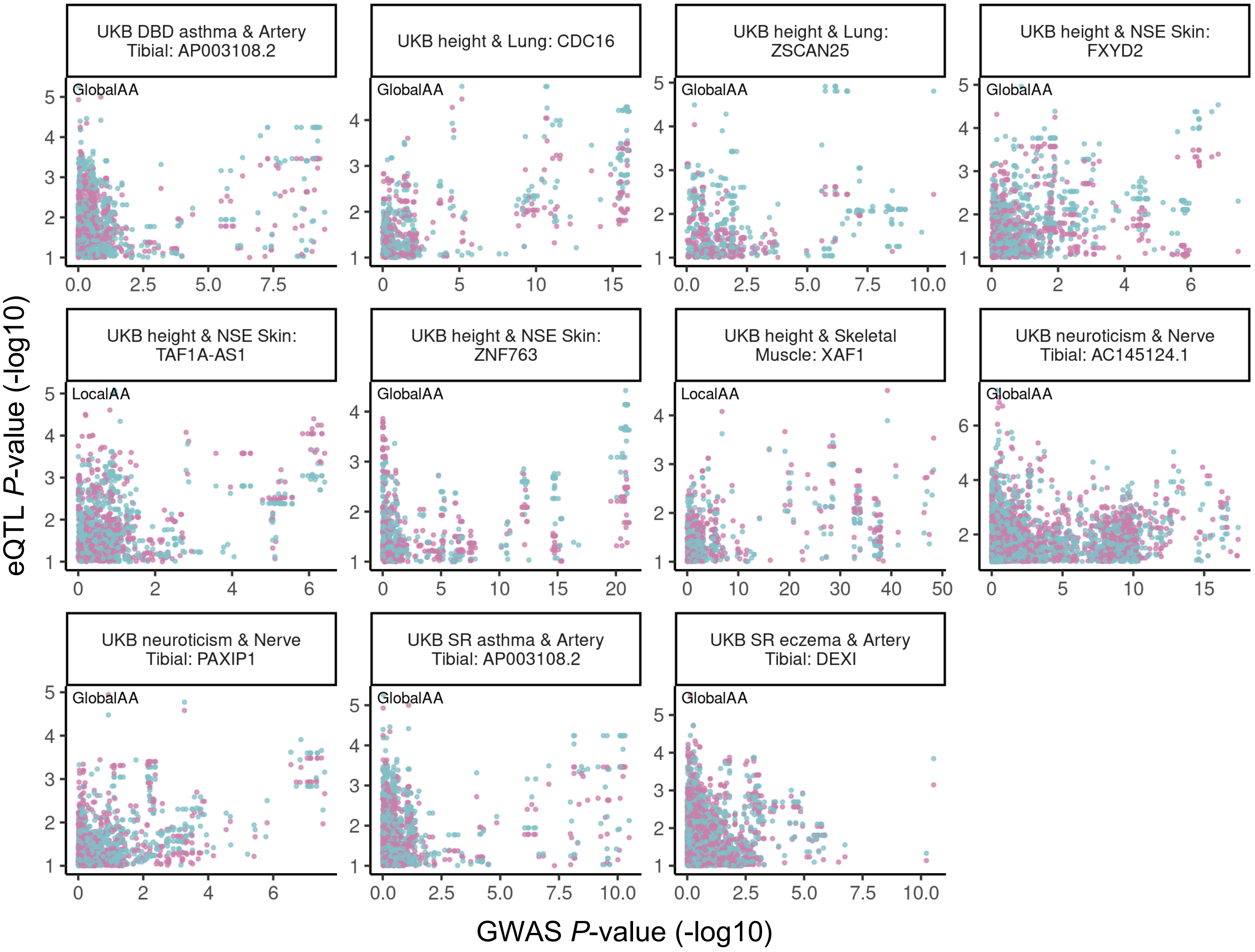

Figure S5. Loci with stronger GWAS colocalizations using one eQTL ancestry adjustment method. A colocalization is considered stronger with Method A than Method B if: 1) only Method A has a COLOC PP4 > 0.5; and 2) only Method A has a FINEMAP CLPP > 0.01. These conditions describe 31 loci from the colocalization analyses between LocalAA or GlobalAA eQTLs and 142 GWAS. 22 and 9 colocalizations are stronger with GlobalAA and LocalAA, respectively. The eQTL ancestry adjustment method with stronger colocalization is indicated in the upper left-hand corner of each plot. All overlapping variants between the eQTL study and GWAS within 1MB of the lead GWAS variant are plotted. In general, the shape of the eQTL signals between the two ancestry adjustment methods are similar, but one method has an overall stronger signal. SR = self-reported; DBD = diagnosed by doctor; N = count; FA = fatty acids.
