# Supplementary material for: Impact of admixture and ancestry on eQTL analysis and GWAS colocalization in GTEx
Source: Genome Biol. 2020 Sep 11;21:233. doi: 10.1186/s13059-020-02113-0 (PMC7488497; doi:10.1186/s13059-020-02113-0)
Supplement: Supplementary file 9 — Additional file 9. GTEx Consortium author list. [file 13059_2020_2113_MOESM9_ESM.pdf]

GTEx Consortium

Laboratory and Data Analysis Coordinating Center (LDACC): François Aguet[1], Shankara Anand[1], Kristin G Ardlie[1], Stacey Gabriel[1], Gad Getz[1,30], Aaron Graubert[1], Kane Hadley[1], Robert E Handsaker[32,33,34], Katherine H Huang[1], Seva Kashin[32,33,34], Xiao Li[1], Daniel G MacArthur[33,35], Samuel R Meier[1], Jared L Nedzel[1], Duyen Y Nguyen[1], Ayellet V Segrè[1,17], Ellen Todres[1]

Analysis Working Group (funded by GTEx project grants): François Aguet[1], Shankara Anand[1], Kristin G Ardlie[1], Brunilda Balliu[40], Alvaro N Barbeira[2], Alexis Battle[18,11], Rodrigo Bonazzola[2], Andrew Brown[3,4], Christopher D Brown[24], Stephane E Castel[5,6], Don Conrad[41,42], Daniel J Cotter[29], Nancy Cox[16], Sayantan Das[26], Olivia M de Goede[29], Emmanouil T Dermitzakis[3,27,28], Barbara E Engelhardt[7,8], Eleazar Eskin[43], Tiffany Y Eulalio[44], Nicole M Ferraro[44], Elise Flynn[5,6], Laure Fresard[12], Eric R Gamazon[13,14,15,16], Diego Garrido-Martín[22], Nicole R Gay[29], Gad Getz[1,30], Aaron Graubert[1], Roderic Guigó[22,31], Kane Hadley[1], Andrew R Hamel[17,1], Robert E Handsaker[32,33,34], Yuan He[18], Paul J Hoffman[5], Farhad Hormozdiari[19,1], Lei Hou[45,1], Katherine H Huang[1], Hae Kyung Im[2], Brian Jo[7,8], Silva Kasela[5,6], Seva Kashin[32,33,34], Manolis Kellis[45,1], Sarah Kim-Hellmuth[5,6,9], Alan Kwong[26], Tuuli Lappalainen[5,6], Xiao Li[1], Xin Li[12], Yanyu Liang[2], Daniel G MacArthur[33,35], Serghei Mangul[43,46], Samuel R Meier[1], Pejman Mohammadi[5,6,20,21], Stephen B Montgomery[12,29], Manuel Muñoz-Aguirre[22,23], Daniel C Nachun[12], Jared L Nedzel[1], Duyen Y Nguyen[1], Andrew B Nobel[47], Meritxell Oliva[2,10], YoSon Park[24,25], Yongjin Park[45,1], Princy Parsana[11], Ferran Reverter[48], John M Rouhana[17,1], Chiara Sabatti[49], Ashis Saha[11], Ayellet V Segrè[1,17], Andrew D Skol[2,50], Matthew Stephens[36], Barbara E Stranger[2,37], Benjamin J Strober[18], Nicole A Teran[12], Ellen Todres[1], Ana Viñuela[38,3,27,28], Gao Wang[36], Xiaoquan Wen[26], Fred Wright[51], Valentin Wucher[22], Yuxin Zou[39]

Analysis Working Group (not funded by GTEx project grants): Pedro G Ferreira[52,53,54], Gen Li[55], Marta Melé[56], Esti Yeger-Lotem[57,58]

Leidos Biomedical - Project Management: Mary E Barcus[59], Debra Bradbury[60], Tanya Krubit[60], Jeffrey A McLean[60], Liqun Qi[60], Karna Robinson[60], Nancy V Roche[60], Anna M Smith[60], Leslie Sobin[60], David E Tabor[60], Anita Undale[60]

Biospecimen collection source sites: Jason Bridge[61], Lori E Brigham[62], Barbara A Foster[63], Bryan M Gillard[63], Richard Hasz[64], Marcus Hunter[65], Christopher Johns[66], Mark Johnson[67], Ellen Karasik[63], Gene Kopen[68], William F Leinweber[68], Alisa McDonald[68], Michael T Moser[63], Kevin Myer[65], Kimberley D Ramsey[63], Brian Roe[65], Saboor Shad[68], Jeffrey A Thomas[68,67], Gary Walters[67], Michael Washington[67], Joseph Wheeler[66]

Biospecimen core resource: Scott D Jewell[69], Daniel C Rohrer[69], Dana R Valley[69]

Brain bank repository: David A Davis[70], Deborah C Mash[70]

Pathology: Mary E Barcus[59], Philip A Branton[71], Leslie Sobin[60]

ELSI study: Laura K Barker[72], Heather M Gardiner[72], Maghboeba Mosavel[73], Laura A Siminoff[72]

Genome Browser Data Integration & Visualization: Paul Flicek[74], Maximilian Haeussler[75], Thomas Juettemann[74], W James Kent[75], Christopher M Lee[75], Conner C Powell[75], Kate R Rosenbloom[75], Magali Ruffier[74], Dan Sheppard[74], Kieron Taylor[74], Stephen J Trevanion[74], Daniel R Zerbino[74]

eGTEx groups: Nathan S Abell[29], Joshua Akey[76], Lin Chen[10], Kathryn Demanelis[10], Jennifer A Doherty[77], Andrew P Feinberg[78], Kasper D Hansen[79], Peter F Hickey[80], Lei Hou[45,1], Farzana Jasmine[10], Lihua Jiang[29], Rajinder Kaul[81,82], Manolis Kellis[45,1], Muhammad G Kibriya[10], Jin Billy Li[29], Qin Li[29], Shin Lin[83], Sandra E Linder[29], Stephen B Montgomery[12,29], Meritxell Oliva[2,10], Yongjin Park[45,1], Brandon L Pierce[10], Lindsay F Rizzardi[84], Andrew D Skol[2,50], Kevin S Smith[12], Michael Snyder[29], John Stamatoyannopoulos[81,85], Barbara E Stranger[2,37], Hua Tang[29], Meng Wang[29]

NIH program management: Philip A Branton[71], Latarsha J Carithers[71,86], Ping Guan[71], Susan E Koester[87], A. Roger Little[88], Helen M Moore[71], Concepcion R Nierras[89], Abhi K Rao[71], Jimmie B Vaught[71], Simona Volpi[90]

Affiliations

1. The Broad Institute of MIT and Harvard, Cambridge, MA, USA
2. Section of Genetic Medicine, Department of Medicine, The University of Chicago, Chicago, IL, USA
3. Department of Genetic Medicine and Development, University of Geneva Medical School, Geneva, Switzerland

4. Population Health and Genomics, University of Dundee, Dundee, Scotland, UK
5. New York Genome Center, New York, NY, USA
6. Department of Systems Biology, Columbia University, New York, NY, USA
7. Department of Computer Science, Princeton University, Princeton, NJ, USA
8. Center for Statistics and Machine Learning, Princeton University, Princeton, NJ, USA
9. Statistical Genetics, Max Planck Institute of Psychiatry, Munich, Germany
10. Department of Public Health Sciences, The University of Chicago, Chicago, IL, USA
11. Department of Computer Science, Johns Hopkins University, Baltimore, MD, USA
12. Department of Pathology, Stanford University, Stanford, CA, USA
13. Data Science Institute, Vanderbilt University, Nashville, TN, USA
14. Clare Hall, University of Cambridge, Cambridge, UK
15. MRC Epidemiology Unit, University of Cambridge, Cambridge, UK
16. Division of Genetic Medicine, Department of Medicine, Vanderbilt University Medical Center, Nashville, TN, USA
17. Ocular Genomics Institute, Massachusetts Eye and Ear, Harvard Medical School, Boston, MA, USA
18. Department of Biomedical Engineering, Johns Hopkins University, Baltimore, MD, USA
19. Department of Epidemiology, Harvard T.H. Chan School of Public Health, Boston, MA, USA
20. Scripps Research Translational Institute, La Jolla, CA, USA
21. Department of Integrative Structural and Computational Biology, The Scripps Research Institute, La Jolla, CA, USA
22. Centre for Genomic Regulation (CRG), The Barcelona Institute for Science and Technology, Barcelona, Catalonia, Spain
23. Department of Statistics and Operations Research, Universitat Politècnica de Catalunya (UPC), Barcelona, Catalonia, Spain
24. Department of Genetics, University of Pennsylvania, Perelman School of Medicine, Philadelphia, PA, USA
25. Department of Systems Pharmacology and Translational Therapeutics, University of Pennsylvania, Perelman School of Medicine, Philadelphia, PA, USA
26. Department of Biostatistics, University of Michigan, Ann Arbor, MI, USA
27. Institute for Genetics and Genomics in Geneva (iGE3), University of Geneva, Geneva, Switzerland
28. Swiss Institute of Bioinformatics, Geneva, Switzerland
29. Department of Genetics, Stanford University, Stanford, CA, USA
30. Cancer Center and Department of Pathology, Massachusetts General Hospital, Boston, MA, USA
31. Universitat Pompeu Fabra (UPF), Barcelona, Catalonia, Spain
32. Department of Genetics, Harvard Medical School, Boston, MA, USA
33. Program in Medical and Population Genetics, The Broad Institute of Massachusetts Institute of Technology and Harvard University, Cambridge, MA, USA
34. Stanley Center for Psychiatric Research, Broad Institute, Cambridge, MA, USA
35. Analytic and Translational Genetics Unit, Massachusetts General Hospital, Boston, MA, USA
36. Department of Human Genetics, University of Chicago, Chicago, IL, USA
37. Center for Genetic Medicine, Department of Pharmacology, Northwestern University, Feinberg School of Medicine, Chicago, IL, USA
38. Department of Twin Research and Genetic Epidemiology, King's College London, London, UK
39. Department of Statistics, University of Chicago, Chicago, IL, USA
40. Department of Biomathematics, University of California, Los Angeles, Los Angeles, CA, USA
41. Department of Genetics, Washington University School of Medicine, St. Louis, Missouri, USA
42. Department of Pathology & Immunology, Washington University School of Medicine, St. Louis, Missouri, USA
43. Department of Computer Science, University of California, Los Angeles, Los Angeles, CA, USA
44. Program in Biomedical Informatics, Stanford University School of Medicine, Stanford, CA, USA

45. Computer Science and Artificial Intelligence Laboratory, Massachusetts Institute of Technology, Cambridge, MA, USA
46. Department of Clinical Pharmacy, School of Pharmacy, University of Southern California, Los Angeles, CA, USA
47. Department of Statistics and Operations Research and Department of Biostatistics, University of North Carolina, Chapel Hill, NC, USA
48. Department of Genetics, Microbiology and Statistics, University of Barcelona, Barcelona. Spain.
49. Departments of Biomedical Data Science and Statistics, Stanford University, Stanford, CA, USA
50. Department of Pathology and Laboratory Medicine, Ann & Robert H. Lurie Children's Hospital of Chicago, Chicago, IL, USA
51. Bioinformatics Research Center and Departments of Statistics and Biological Sciences, North Carolina State University, Raleigh, NC, USA
52. Department of Computer Sciences, Faculty of Sciences, University of Porto, Porto, Portugal
53. Instituto de Investigação e Inovação em Saúde, Universidade do Porto, Porto, Portugal
54. Institute of Molecular Pathology and Immunology, University of Porto, Porto, Portugal
55. Columbia University Mailman School of Public Health, New York, NY, USA
56. Life Sciences Department, Barcelona Supercomputing Center, Barcelona, Spain
57. Department of Clinical Biochemistry and Pharmacology, Ben-Gurion University of the Negev, Beer-Sheva, Israel
58. National Institute for Biotechnology in the Negev, Beer-Sheva, Israel
59. Leidos Biomedical, Frederick, MD, USA
60. Leidos Biomedical, Rockville, MD, USA
61. UNYTS, Buffalo, NY, USA
62. Washington Regional Transplant Community, Annandale, VA, USA
63. Therapeutics, Roswell Park Comprehensive Cancer Center, Buffalo, NY, USA
64. Gift of Life Donor Program, Philadelphia, PA, USA
65. LifeGift, Houston, TX, USA
66. Center for Organ Recovery and Education, Pittsburgh, PA, USA
67. LifeNet Health, Virginia Beach, VA. USA
68. National Disease Research Interchange, Philadelphia, PA, USA
69. Van Andel Research Institute, Grand Rapids, MI, USA
70. Department of Neurology, University of Miami Miller School of Medicine, Miami, FL, USA
71. Biorepositories and Biospecimen Research Branch, Division of Cancer Treatment and Diagnosis, National Cancer Institute, Bethesda, MD, USA
72. Temple University, Philadelphia, PA, USA
73. Virgina Commonwealth University, Richmond, VA, USA
74. European Molecular Biology Laboratory, European Bioinformatics Institute, Hinxton, United Kingdom
75. Genomics Institute, UC Santa Cruz, Santa Cruz, CA, USA
76. Carl Icahn Laboratory, Princeton University, Princeton, NJ, USA
77. Department of Population Health Sciences, The University of Utah, Salt Lake City, Utah, USA
78. Schools of Medicine, Engineering, and Public Health, Johns Hopkins University, Baltimore, MD, USA
79. Department of Biostatistics, Bloomberg School of Public Health, Johns Hopkins University, Baltimore, MD, USA
80. Department of Medical Biology, The Walter and Eliza Hall Institute of Medical Research, Parkville, Victoria, Australia
81. Altius Institute for Biomedical Sciences, Seattle, WA, USA
82. Division of Genetics, University of Washington, Seattle, WA, University of Washington, Seattle, WA, USA
83. Department of Cardiology, University of Washington, Seattle, WA, USA
84. HudsonAlpha Institute for Biotechnology, Huntsville, AL, USA
85. Genome Sciences, University of Washington, Seattle, WA, USA
86. National Institute of Dental and Craniofacial Research, Bethesda, MD, USA

87. Division of Neuroscience and Basic Behavioral Science, National Institute of Mental Health, National Institutes of Health, Bethesda, MD, USA
88. National Institute on Drug Abuse, Bethesda, MD, USA
89. Office of Strategic Coordination, Division of Program Coordination, Planning and Strategic Initiatives, Office of the Director, National Institutes of Health, Rockville, MD, USA
90. Division of Genomic Medicine, National Human Genome Research Institute, Bethesda, MD, USA
