# Supplementary material for: Impact of admixture and ancestry on eQTL analysis and GWAS colocalization in GTEx
Source: Genome Biol. 2020 Sep 11;21:233. doi: 10.1186/s13059-020-02113-0 (PMC7488497; doi:10.1186/s13059-020-02113-0)
Supplement: Supplementary file 10 — Additional file 10. Review History. [file 13059_2020_2113_MOESM10_ESM.docx]

**Review history**

**First round of review**

**Reviewer 1**

In the paper "Impact of admixture on eQTL analysis and GWAS colocalization in GTEx" the authors ask the important question of if LA adjusted eQTL mapping affect the colocalization to GWAS and hence improves our understanding of the biological mechanisms underlying these associations. I believe the authors have done a good job in trying to answer these questions with the analysis done. However there remains a few points of clarification below which may affect how the data is interpreted.

Major Comments:
1)     The authors present work that is meant to be as large scale as possible utilizing the newest version of GTEx.  Understandably only a few tissues will have sufficient patients to conduct this analysis given the restriction to only admixed individuals. Figure 1b shows the tissue selected based on number of admixed individuals in each tissue dataset (N>60) and "well-studied traits of relevance".  But the authors skip over Whole Blood but include NSE skin. These choices seem arbitrary and really do not seem to correlate trait relevance.
2)     In the methods the author state that 114 GWAS were selected for the colocalization studies. Given that the traits in these studies substantially effect how well eQTLs colocalize, some rational for this choice is needed.
3)     In the comparison on the number of eGenes per tissue for each adjustment method (figure 2b) the authors use a nominal p-value cut off of 1e-6.  Why this as oppose to a true FDR cutoff to better estimate the actual power of each methods corrected for multiple testing. The authors state this p-value approximates FDR, but it seems more straightforward to calculate FDR for these associations.
4)     In the colocalization analysis the authors us COLOC to determine if the method specific eQTLs and GWAS share the same causal variant.  Methods such as COLOC do not take LD differences between GWAS study and eQTL cohort into account when determining shared causal variants. This seems particularly relevant given the admixed ancestry of the eQTL cohort and the overwhelmingly European ancestry of the GWAS catalog (though I am not sure if that is true for the 114 studies as they are not well annotated in the supplementary table).  Hence lack of colocalization (or potential apparent colocalization) may stem from these differences. Would a method such as eCAVIER be more appropriate?
5)     The finding that the Fst_within had larger coefficient than Fst_between is really puzzling and really should be discussed. This seems really unexpected and it would be useful to get an idea on why this would be - at least in the discussion of the paper. Could this be a consequence of using COLOC and the LD issue above?

Minor Comments:
1)     In Figure 3a some but not all the COLOC signals in the pink and green boxes are highlighted. This honestly makes the figure a bit cluttered. It is also hard to garner any real meaning as many of the trait/gene pairs do not really make sense with the tissue they are found in (e.g. any of the tissues and standing height) while others are really well matched (e.g. ezema and skin).   This could be improved with just a few key examples since not all of the dots are expanded and a complete list can be added as supplementary.
2)     I am confused as to why there are more than the original 6 tissues in figure 4? Where the same number of tissues used in the analysis in Figure 1e? I understand that eQTLs were not mapped in these tissues, but they seem arbitrarily added in this analysis and not others.

**Reviewer 2**

This is a really well written paper that was easy to follow, and which addressed an important aspect of GWAS fine-mapping when integrating results with GTEx eQTLs, that has not been well described previously. My comments are mostly minor.

Major comment
A brief rationale for the basis of the selection of 114 GWAS should be added to the main text of the manuscript (the only reference to it that I could find is on line 577), perhaps together with an ancestry breakdown of those 114 GWAS so that it is clear whether there are some multi-ethnic and African ancestry GWAS included, as the results of this paper may be more relevant to those types of GWAS. From a brief glance of the figures in which specific GWAS are listed, many seem to be from the UK Biobank or European ancestry. Perhaps pick one large robust multi-ethnic GWAS with a fairly large proportion of African ancestry participants, to show case that co-localization is not really changed by local ancestry adjustment, or admit to this being a limitation of the study (or explain why it isn't a limitation of the study).

Minor comment
- Line 79: suggest replacing "lead to confounding" with "confound:
- Line 104: suggest replacing N (which people can read as sample size) with M
- Suggest moving Figure 1C to the supplementary
- Figure 1e legend: colors correspond to b, not a?
- Figure 3C is not entirely clear - is it the proportion of variants that reach maximum within Fst as alluded to lines 237-239? This is not clear from the figure legend
- Line 320: "in fact driven by" should be "in fact be driven by"
- Line 447: clarify/reword "type of ancestry" - can be read as European vs African but I think this refers to global vs local ancestry adjustment

**Reviewer 3**

This is a well conducted analysis and a well written paper, on a topic of great importance for understanding complex trait genetics across global populations.
A few mostly minor comments:
The authors do a nice job discussing power for eQTL mapping within the admixed population subset of GTEX v8. However, it would also be nice to know if the LA and/or GA adjusted analysis in this admixed population finds any eQTLs (or colocalized eQTLs specifically) missed by the overall eQTL calling pipeline (with a cross population analysis potentially missing associations with effect heterogeneity across populations)? Would there be any advantage to performing an ancestry stratified analysis in terms of discovering new QTLs, or are nearly all eQTLs also found in the pooled ancestry analysis? Further exploration of this issue would be useful for look-up purposes, similar to the provided list of GTEx v8 eVariants that are potentially confounded by LA. Perhaps this issue has already been explored in an earlier paper; if so, that would be helpful to cite.
A fuller discussion of the implications of this work for planning of future eQTL studies would be helpful. For example, the authors could discuss the need to include other admixed populations, such as Hispanics, in eQTL analyses, the potential for identification of additional eQTL variants strongly correlated with ancestry background as ancestrally diverse sample sizes increase, and the increased computational complexity of local ancestry adjustment in study populations containing individuals with admixture from a greater number of continental ancestral groups.
In the results, by "well-studied trait relevance (20-24)", do you mean that these tissues are relevant to complex phenotypes that vary across race/ethnicity groups? This wording should be clarified, so readers can better understand why these tissues were selected. For example, why not include whole blood in your analyses?
For the AP3S2 GWAS signal, do the lead variants greatly differ in frequency between global populations? In which population are they most common? Is this why the authors pointed out that the GWAS signals were identified in diverse populations?
For the colocalization analysis, it doesn't look like any effort was made to look at colocalization of secondary signals using approximate conditional analysis or other methods (as described in <https://www.biorxiv.org/content/10.1101/838946v2>). This should perhaps be mentioned as a limitation.
Throughout, you should clarify that you are referring to East Asian, not South Asian populations (based on the 1000G populations included in the methods section).
Was the 'cc' trait type parameters used for the COLOC analyses or not? I find this confusing as written.

**Authors’ response to reviewers**

Reviewer #1

Comment: In the paper "Impact of admixture on eQTL analysis and GWAS

colocalization in GTEx" the authors ask the important question of if LA adjusted eQTL

mapping affect the colocalization to GWAS and hence improves our understanding of

the biological mechanisms underlying these associations. I believe the authors have

done a good job in trying to answer these questions with the analysis done.

*Response: Thank you for the supportive comment.*

Major Comments

Comment: The authors present work that is meant to be as large scale as possible

utilizing the newest version of GTEx. Understandably only a few tissues will have

sufficient patients to conduct this analysis given the restriction to only admixed individuals. Figure 1b shows the tissue selected based on number of admixed individuals in each tissue dataset (N>60) and "well-studied traits of relevance". But the authors skip over Whole Blood but include NSE skin. These choices seem arbitrary and really do not seem to correlate trait relevance.

*Response: We originally excluded Whole Blood because GTEx whole blood often*

*separates in PC space from whole blood profiles collected from living donors in other*

*studies. However, this does not preclude identifying important eGenes in GTEx whole*

*blood. We have now performed LocalAA and GlobalAA eQTL calling in Whole Blood*

*and incorporated these results into all downstream analyses in the manuscript. Other*

*tissues were selected based on a combination of adequate sample size (e.g. Tibial*

*Nerve) and relevance to phenotypes with known population differences. We have now*

*clarified this in the main text:*

*“We selected seven tissues in which to perform cis-eQTL calling based on a minimum*

*admixed sample size of 60 (20) and relevance to phenotypes with known population*

*differences (e.g. subcutaneous adipose and body fat distribution (21,22), N=84; notsun-*

*exposed (NSE) skin and epidermal gene expression (23), N=71; lung and asthma*

*prevalence (24), N=64; skeletal muscle and lean muscle mass (25), N=98. Whole*

*blood (N=95) and tibial artery (N=89) were also included because they have large*

*117AX sample sizes.”*

Comment: In the methods the author state that 114 GWAS were selected for the

colocalization studies. Given that the traits in these studies substantially affect how well

eQTLs colocalize, some rationale for this choice is needed.

*Response: The original 114 GWAS were chosen to include a broad representation of*

*different trait classes and some replication between GWAS from UK BioBank and other*

*consortia. We have now clarified this in the main text and added a supplementary table*

*with more information about each GWAS, i.e. the original 114 GWAS and an additional*

*28 GWAS from the multi-ethnic PAGE study.*

*We now add:*

*Background*

*“For the subset of loci where the two ancestry adjustment methods yield different*

*results, we perform GWAS/eQTL colocalization analyses with 142 previously published*

*GWAS, representing a range of traits, consortia, and cohort ancestry.”*

*Results*

*“We selected 142 GWAS to perform colocalization with our eQTLs. Previously, 114 of*

*these GWAS were used to perform colocalization with all GTEx v8 eQTLs (33). These*

*GWAS were originally chosen to include a broad representation of different trait*

*classes and some replication between GWAS from UK BioBank (UKB) and other*

*consortia. We included an additional 28 multiethnic GWAS from the PAGE study to*

*increase the representation of admixed cohorts in our colocalization analyses (34).*

*More information about each GWAS is available in Table S5 (Additional file 2).”*

Comment: In the comparison on the number of eGenes per tissue for each adjustment

method (figure 2b) the authors use a nominal p-value cut off of 1e-6. Why this as

opposed to a true FDR cutoff to better estimate the actual power of each method

corrected for multiple testing. The authors state this p-value approximates FDR, but it

seems more straightforward to calculate FDR for these associations.

*Response: Adjustment for multiple testing is challenging in the context of regressions*

*with variant-specific covariates (i.e. local ancestry covariates). A straightforward genelevel*

*Bonferroni correction followed by a genome-wide BH adjustment is prohibitively*

*conservative, particularly with our small sample sizes of admixed individuals in these*

*tissues. Permutation procedures to generate null distributions of lead P-values are*

*often used as a less conservative approach to generate gene-level adjusted P-values*

*(ref FastQTL). For example, the GTEx Consortium obtained gene-level q-values using*

*FastQTL’s beta distribution-extrapolated empirical P-values. This involves repeating*

*the cis-eQTL calling procedure for some minimal number of permutations R of each*

*gene’s expression, using maximum likelihood estimation to fit the resulting R lead Pvalues*

*to a beta distribution for that gene, and defining the adjusted P-value as the*

*probability of observing a P-value smaller than the true nominal P-value from the fitted*

*beta distribution. This is much less computationally intensive than a direct or adaptive*

*permutation procedure to obtain adjusted P-values, which can require upwards of*

*100,000 iterations of cis-eQTL calling per gene. However, even for R=100, which is a*

*modest number of permutations, calculating beta-approximated P-values for the genes*

*in all 7 eQTL tissues would require more than 186 trillion linear regressions. With the*

*computational resources available to us, that equates to 1,082,594 CPU hours, or a*

*$54,000 bill. To demonstrate the correlation between a nominal P-value cutoff of 1e-06*

*and a 5% FDR, we performed the beta approximation for a subset of genes on*

*chromosome 22 in 3 of the eQTL tissues (Lung, Skeletal Muscle, Subcutaneous*

*Adipose). We added a supplementary figure (Figure S4, Additional file 1) that shows*

*the relationship between nominal P-values and gene-level beta-approximated P-values*

*and Storey’s qvalues for those beta-approximated P-values; a nominal P-value of 10e-*

*06 corresponds with a qvalue of 0.045 for these loci. We also recognize the*

*computational intractability of permutation procedures as a limitation of local ancestry*

*adjustment.*

Comment: In the colocalization analysis the authors use COLOC to determine if the

method specific eQTLs and GWAS share the same causal variant. Methods such as

COLOC do not take LD differences between GWAS study and eQTL cohort into

account when determining shared causal variants. This seems particularly relevant

given the admixed ancestry of the eQTL cohort and the overwhelmingly European

ancestry of the GWAS catalog (though I am not sure if that is true for the 114 studies

as they are not well annotated in the supplementary table). Hence lack of

colocalization (or potential apparent colocalization) may stem from these differences.

Would a method such as eCAVIER be more appropriate?

*Response: Thank you for this suggestion. You are correct, the vast majority of the*

*original 114 GWAS have primarily European cohorts. We have now included an*

*additional 28 GWAS from the PAGE study, with a cohort of 49,839 non-European*

*individuals (Wojcik et al., Nature, 2019). We also added a supplementary table with*

*better annotation of the populations in each GWAS (Table S5, Additional file 2). For all*

*tested loci with a COLOC PP4 > 0.5 in either eQTL adjustment method, we have now*

*performed colocalization with an implementation of FINEMAP, which is similar to*

*eCAVIAR. Figure 3 now includes results from colocalization analyses with both*

*COLOC and FINEMAP; we added notation of European versus multi-ethnic GWAS*

*cohorts to aid interpretation. From loci in the PAGE GWAS, no LocalAA eQTLs*

*consistently colocalize more strongly than GlobalAA eQTLs or vice versa.*

Comment: The finding that the Fst_within had larger coefficient than Fst_between is

really puzzling and really should be discussed. This seems really unexpected and it

would be useful to get an idea on why this would be - at least in the discussion of the

paper. Could this be a consequence of using COLOC and the LD issue above?

*Response: We agree. To your point, we thought that incorporating multi-ethnic GWAS*

*could help illuminate this result. However, after including the PAGE GWAS and*

*repeating the analysis, the results were no more intuitive. We tried implementing a*

*linear mixed model to predict colocalization probabilities instead; this model allowed us*

*to include all observations in a single regression, which made the coefficient estimates*

*more interpretable. However, the results were still puzzling. Therefore, we have*

*decided to remove the FST analysis from the manuscript. This comprised a small*

*section of the paper and was never a major focus of the study. In the original*

*manuscript, we also acknowledged that the result of the regression was not what we*

*expected. We have instead expanded on results from the newly added PAGE and*

*FINEMAP colocalization analyses.*

Minor Comments

Comment: In Figure 3a some but not all the COLOC signals in the pink and green

boxes are highlighted. This honestly makes the figure a bit cluttered. It is also hard to

garner any real meaning as many of the trait/gene pairs do not really make sense with

the tissue they are found in (e.g. any of the tissues and standing height) while others

are really well matched (e.g. eczema and skin). This could be improved with just a

few key examples since not all of the dots are expanded and a complete list can be

added as supplementary.

*Response: Thank you for this recommendation. Fig 3a did look cluttered. Instead of*

*labelling all points that had stronger COLOC colocalizations with either LocalAA or*

*GlobalAA, we now highlight the 31 loci where one eQTL ancestry adjustment method*

*has substantially stronger colocalizations as reported by both COLOC and FINEMAP.*

*Figure 3c and Figure S5 further characterize these loci; Table S6 includes all of the*

*colocalization results.*

Comment: I am confused as to why there are more than the original 6 tissues in figure

4? Were the same number of tissues used in the analysis in Figure 1e? I understand

that eQTLs were not mapped in these tissues, but they seem arbitrarily added in this

analysis and not others.

*Response: Figure 1e and Figure 4 have some important differences. Figure 1e*

*includes only 117AX (i.e. gene expression and local/global ancestry covariates in the*

*admixed subcohort), and it assesses variance in residual expression explained by local*

*or global ancestry. Figure 4 instead looks at variance in eVariant genotype (not gene*

*expression) explained by local ancestry, with eVariants being defined as variants*

*associated with all significant associations reported by GTEx v8. Therefore, Figure 4*

*also includes genotype and local ancestry information for all 838 genotyped individuals*

*in the GTEx v8 cohort, not just 117AX. Since sample sizes differ between the 49 GTEx*

*v8 tissues, we performed the analysis with samples matched to those with gene*

*expression in each tissue. This is why there are additional tissues in Figure 4 relative to*

*the rest of the manuscript. Because the consortium performed COLOC colocalization*

*analyses between eQTL summary statistics for all 49 tissues and 114 imputed GWAS*

*summary statistics, we also annotated eQTLs in Figure 4 that are associated with*

*putative colocalizations (COLOC PP4 > 0.5). These colocalizations are from an*

*analysis entirely independent of the analysis used to generate Figure 3a. The*

*motivation for Figure 4 was to generate a resource of gene-variant pairs reported by*

*the consortium as having a significant association that are potentially confounded by*

*local ancestry.This information is more broadly relevant to individuals using GTEx v8*

*data than the related analysis restricted to 117AX (Figure 1e). We tried to make this*

*more clear and made the following additions. Please let us know if it is still unclear.*

*Figure 4 legend*

*“For all eVariants reported by the overall GTEx v8 eQTL calling pipeline, we calculated*

*the correlation between genotypes and local ancestry using the full GTEx v8 cohort.”*

*Results*

*“We examined all significant associations reported by the overall GTEx v8 eQTL calling*

*pipeline for evidence of confounding with LA. Note that this analysis is expanded to*

*include the full GTEx v8 cohort, not just the admixed sub-cohort involved in preceding*

*analyses.”*

Reviewer #2

Comment: This is a really well written paper that was easy to follow, and which

addressed an important aspect of GWAS fine-mapping when integrating results with

GTEx eQTLs, that has not been well described previously. My comments are mostly

minor.

*Response: Thank you for the supportive comments.*

Comment: A brief rationale for the basis of the selection of 114 GWAS should be

added to the main text of the manuscript (the only reference to it that I could find is on

line 577), perhaps together with an ancestry breakdown of those 114 GWAS so that it

is clear whether there are some multi-ethnic and African ancestry GWAS included, as

the results of this paper may be more relevant to those types of GWAS. From a brief

glance of the figures in which specific GWAS are listed, many seem to be from the UK

Biobank or European ancestry. Perhaps pick one large robust multi-ethnic GWAS with

a fairly large proportion of African ancestry participants, to show case that colocalization

is not really changed by local ancestry adjustment, or admit to this being a

limitation of the study (or explain why it isn't a limitation of the study).

*Response: You are correct, the vast majority of the original 114 GWAS have primarily*

*European cohorts. We have now included an additional 28 GWAS from the PAGE*

*study, with a cohort of 49,839 non-European individuals: Hispanic/Latino (n = 22,216),*

*African American (n = 17,299), Asian (n = 4,680), Native Hawaiian (n = 3,940), Native*

*American (n = 652) or Other (n = 1,052) (self-reported ancestry) (Wojcik et al., Nature,*

*2019). Indeed, even with these multi-ethnic GWAS, there are no systematic differences*

*in GWAS colocalization between GlobalAA and LocalAA eQTLs. We added notation of*

*European versus multi-ethnic GWAS cohorts to Figure 3a-b to aid interpretation. We*

*also added a supplementary table with better annotation of the populations in each*

*GWAS (Table S5, Additional file 2). The original 114 GWAS were chosen to have a*

*broad representation of different trait classes as well as some replication between*

*GWAS from UK BioBank and other consortia. We have now clarified this in the main*

*text:*

*Background*

*“For the subset of loci where the two ancestry adjustment methods yield different*

*results, we perform GWAS/eQTL colocalization analyses with 142 previously published*

*GWAS, representing a range of traits, consortia, and cohort ancestry.”*

*Results*

*“We selected 142 GWAS to perform colocalization with our eQTLs. Previously, 114 of*

*these GWAS were used to perform colocalization with all GTEx v8 eQTLs (33). These*

*GWAS were originally chosen to include a broad representation of different trait*

*classes and some replication between GWAS from UK BioBank (UKB) and other*

*consortia. We included an additional 28 multiethnic GWAS from the PAGE study to*

*increase the representation of admixed cohorts in our colocalization analyses (34).*

*More information about each GWAS is available in Table S5 (Additional file 2).”*

Minor comments

Line 79: suggest replacing "lead to confounding" with "confound”

Line 104: suggest replacing N (which people can read as sample size) with M

Figure 1e legend: colors correspond to b, not a?

Line 320: "in fact driven by" should be "in fact be driven by"

Line 447: clarify/reword "type of ancestry" - can be read as European vs African but I

think this refers to global vs local ancestry adjustment

Response: Thank you for these suggestions and corrections! We made these changes.

Comment: Suggest moving Figure 1C to the supplementary

*Response: We truncated Fig 1C to include only 4 chromosomes. We would like to*

*retain this in a main figure to provide a visual representation of local ancestry and the*

*degree of admixture in our cohort. Figure S3 does include a similar plot for all*

*autosomal chromosomes.*

Comment: Figure 3C is not entirely clear - is it the proportion of variants that reach

maximum within Fst as alluded to lines 237-239? This is not clear from the figure

legend

*Response: Yes, this is the proportion of variants where the maximum of the 20*

*pairwise Fst calculations corresponds with the given pair of populations. However, we*

*have decided to remove the FST analysis from the manuscript (refer to Reviewer #1*

*Major Comments).*

Reviewer #3

Comment: This is a well conducted analysis and a well written paper, on a topic of

great importance for understanding complex trait genetics across global populations.

Response: Thank you for the supportive comments.

Comment: The authors do a nice job discussing power for eQTL mapping within the

admixed population subset of GTEX v8. However, it would also be nice to know if the

LA and/or GA adjusted analysis in this admixed population finds any eQTLs (or

colocalized eQTLs specifically) missed by the overall eQTL calling pipeline (with a

cross population analysis potentially missing associations with effect heterogeneity

across populations)? Would there be any advantage to performing an ancestry

stratified analysis in terms of discovering new QTLs, or are nearly all eQTLs also found

in the pooled ancestry analysis? Further exploration of this issue would be useful for

look-up purposes, similar to the provided list of GTEx v8 eVariants that are potentially

confounded by LA. Perhaps this issue has already been explored in an earlier paper; if

so, that would be helpful to cite.

*Response: Thank you for this comment. Although the global ancestry adjustment*

*implemented in this manuscript is statistically identical to the overall GTEx v8 eQTL*

*calling pipeline with FastQTL, of the 2882 GlobalAA eGenes across 7 tissues, 261*

*eGenes do not replicate in the GTEx v8 results at a nominal P-value threshold of 1e-6.*

*Similarly, of the 3057 LocalAA eGenes, 270 do not replicate in the GTEx v8 results at a*

*nominal P-value threshold of 1e-6. However, because there are differences between*

*GTEx v8 eQTL calling and our eQTL calling beyond cohort ancestry (sample sizes and*

*both number and composition of PEER factors included as covariates to adjust for*

*hidden variation), we cannot say that the discrepancies between LocalAA/GlobalAA*

*and GTEx are solely a product of differences in cohort ancestry. While populationstratified*

*eQTL calling has not been performed for GTEx v8 to our knowledge, the*

*consortium did characterize population-biased eQTLs; they discovered 178 pb-eQTLs*

*for 141 unique eGenes across 31 tissues at a false discovery rate of 25%. Populationstratified*

*eQTL calling has been performed in other cohorts, namely MESA (Mogil et*

*al., PLoS Genet, 2018); this analysis is very relevant in the context of admixed cohorts,*

*so thank you for asking us to think more about this. We have added the following*

*comments and references to the discussion:*

*“Population-stratified eQTL calling is another potential approach in heterogeneous*

*cohorts. To our knowledge, population-stratified eQTL calling has not yet been*

*performed in the GTEx v8 cohort. However, the consortium did characterize*

*population-biased cis-eQTLs (pb-eQTLs), where a variant’s molecular effect on gene*

*expression differs between individuals of European and African ancestry (Aguet et al,*

*bioRxiv, 2019). Only 178 pb-eQTLs for 141 unique eGenes (FDR ≤ 25%) were*

*identified across 31 tissues, which indicates that pb-eQTLs are hard to find and*

*generally have small effects. Relatedly, Mogil et al. performed population-stratified*

*eQTL calling independently in African American, Hispanic American, and European*

*American samples in MESA; among several replication cohorts, the highest replication*

*rate for all three discovery populations was in the Framingham Heart Study, a*

*European cohort, simply because the sample size was much larger than the other*

*population-matched replication cohorts (Mogil et al., PLoS Genet, 2018). This result,*

*combined with the paucity of eQTLs with robust differences in effect sizes between*

*populations, suggests that population-stratified eQTL calling at current sample sizes is*

*limited in its ability to discover eQTLs not found in a pooled analysis.”*

Comment: A fuller discussion of the implications of this work for planning of future

eQTL studies would be helpful. For example, the authors could discuss the need to

include other admixed populations, such as Hispanics, in eQTL analyses, the potential

for identification of additional eQTL variants strongly correlated with ancestry

background as ancestrally diverse sample sizes increase, and the increased

computational complexity of local ancestry adjustment in study populations containing

individuals with admixture from a greater number of continental ancestral groups.

*Response: Thank you for these specific suggestions. We added comments to the*

*Discussion related to the benefits of including admixed populations in genetic studies*

*and challenges associated with larger reference panels for local ancestry inference.*

*Indeed, we have heard there might be effort from the NIH to expand representation in*

*studies like GTEx and think there is considerable need to expand to Hispanics and*

*other ethnicities.*

*We now include:*

*“One limitation of local ancestry inference is its dependence on the availability of*

*appropriate reference panels. Access to genetic data for some populations remains*

*limited, which makes it challenging to estimate local ancestry from those groups*

*(26,46). Even with access to sufficient numbers of reference panels, there is a limit to*

*the resolution that can be achieved with local ancestry inference given that local*

*ancestry becomes more difficult to estimate as the genetic similarity between reference*

*populations increases (11). Addressing these challenges in future, larger functional*

*genomics studies stands to improve our understanding of genetic risk across*

*populations (47,48) and resolution for the identification of causal variants (49).”*

Comment: In the results, by "well-studied trait relevance (20-24)", do you mean that

these tissues are relevant to complex phenotypes that vary across race/ethnicity

groups? This wording should be clarified, so readers can better understand why these

tissues were selected. For example, why not include whole blood in your analyses?

*Response: Yes, we meant that these tissues are relevant to traits that have known*

*population differences. We originally excluded Whole Blood because GTEx whole*

*blood often separates in PC space from whole blood profiles collected from living*

*donors in other studies. However, this does not preclude identifying important eGenes*

*in GTEx whole blood. We have now performed LocalAA and GlobalAA eQTL calling in*

*Whole Blood and incorporated these results into all downstream analyses in the*

*manuscript. We have now clarified this in the text:*

*“We selected seven tissues in which to perform cis-eQTL calling based on a minimum*

*admixed sample size of 60 (20) and relevance to phenotypes with known population*

*differences (e.g. subcutaneous adipose and body fat distribution (21,22), N=84; notsun-*

*exposed (NSE) skin and epidermal gene expression (23), N=71; lung and asthma*

*prevalence (24), N=64; skeletal muscle and lean muscle mass (25), N=98. Whole*

*blood (N=95) and tibial artery (N=89) were also included because they have large*

*117AX sample sizes.”*

Comment: For the AP3S2 GWAS signal, do the lead variants greatly differ in frequency

between global populations? In which population are they most common? Is this why

the authors pointed out that the GWAS signals were identified in diverse populations?

*Response: Thank you for the suggestion to take a closer look at this locus. Based on*

*1000 Genomes populations, the GlobalAA and LocalAA lead variants have similar*

*differences in allele frequencies across EAS, EUR, and AFR. After performing*

*additional colocalization analyses and finding a lack of differences in colocalization with*

*multi-ethic GWAS, we decided to remove reference to this locus from the text and*

*instead focus on a different subset of more interesting and interpretable loci.*

*We added the following results:*

*“Of 174,388 loci tested for colocalization, 793 loci (<0.5%) have at least one*

*colocalization reported by either COLOC or FINEMAP. Only 159 of these loci have at*

*least one concordant colocalization reported by both COLOC and FINEMAP (i.e. both*

*methods report a colocalization for LocalAA or GlobalAA or both). For a subset of 31*

*loci, one ancestry-adjusted eQTL signal colocalized but the other did not, with*

*concordant results between two colocalization methods. 22 and 9 loci demonstrate*

*stronger colocalization with GlobalAA and LocalAA, respectively (highlighted points,*

*Figure 3a-b; Figure 3c; Figure S5, Additional file 1). Interestingly, all 31 loci correspond*

*with GWAS in primarily European cohorts, regardless of whether colocalization is*

*stronger with GlobalAA or LocalAA.*

*Six of the loci with stronger GlobalAA colocalizations are associated with the same*

*eGene, AP003108.2 in tibial artery. The six colocalized GWAS are associated with*

*three types of traits: asthma (UKB self-reported asthma; UBK diagnosed-by-doctor*

*asthma); red blood cell counts (Astle et al. red blood cell count; Astle et al. reticulocyte*

*count); and fatty acids (GLGC triglycerides; MAGNETIC CH2:double bond ratio in*

*circulating fatty acids). Despite this replicated colocalization, neither the unannotated*

*gene AP003108.2 nor the GlobalAA lead eVariant, rs492751, have reported*

*associations in the GWAS Catalogue (35). We further observed that rs492751 has*

*highly variable allele frequencies between 1000 Genomes superpopulations*

*(alternative allele frequencies of 0.02, 0, and 0.76 in European, East Asian, and African*

*populations, respectively). This suggests that these stronger colocalizations with the*

*GlobalAA tibial artery AP003108.2 eQTL signal may in fact be driven by spurious*

*associations confounded by local ancestry. Notably, a stronger colocalization with one*

*eQTL ancestry adjustment is not synonymous with a more accurate eQTL signal;*

*confounded associations can yield false discoveries.*

*Two loci with stronger LocalAA colocalizations correspond with MYO3A in tibial nerve.*

*The associated traits are eosinophil counts and high light scatter reticulocyte counts*

*(Astle et al.). MYO3A associations with interleukin-6, cortisol secretion, and BMIadjusted*

*waist circumference have previously been reported (35); in other studies,*

*eosinophil counts and characteristics of red blood cells have been correlated with*

*obesity or BMI (36,37), and obesity is associated with an inflammatory response*

*(38,39). Therefore, a true colocalization between the tibial nerve MYO3A eQTL and*

*traits related to properties of immature red blood cells and white blood cells is*

*plausible. This locus provides an example of where LocalAA may outperform GlobalAA*

*in terms of capturing true eQTL signals. However, we acknowledge that the differences*

*in colocalization probabilities are smaller when LocalAA has a stronger colocalization*

*compared to when GlobalAA has a stronger colocalization. In general, LocalAA may*

*reduce false associations more often than it discovers true associations not also*

*identified with GlobalAA. Overall, we observe that neither LocalAA nor GlobalAA*

*performs significantly better in the context of colocalization, regardless of GWAS*

*ancestry or colocalization method.”*

Comment: For the colocalization analysis, it doesn't look like any effort was made to

look at colocalization of secondary signals using approximate conditional analysis or

other methods (as described in https://www.biorxiv.org/content/10.1101/838946v2).

This should perhaps be mentioned as a limitation.

*Response: Thank you for bringing this to our attention. You are entirely correct - we*

*used the assumption of one causal variant per trait and did not attempt to perform*

*colocalization of secondary signals. We added this as a limitation of our analysis and*

*referenced this study:*

*“Limitations of our colocalization analyses include our use of the assumption of one*

*causal variant per trait and our lack of an attempt to colocalize secondary signals*

*(Wallace, 2019; Dobbyn, 2018).”*

Comment: Throughout, you should clarify that you are referring to East Asian, not

South Asian populations (based on the 1000G populations included in the methods

section).

*Response: Thank you, this is an important point of clarification. We changed all*

*relevant instances of “Asian” to say “East Asian”.*

Comment: Was the 'cc' trait type parameters used for the COLOC analyses or not? I

find this confusing as written.

*Response: Originally, the ‘quant’ trait type was specified for all GWAS, including*

*case/control studies. This was an oversight during parallelization of the analysis. We*

*have now repeated COLOC analyses for all case/control studies using ‘cc’ for the trait*

*type parameter and substituted these results. We updated the methods to reflect this*

*change.*

**Second round of review**

**Reviewer 1**

The authors have improved the manuscript and all comments and concerns have been addressed.

**Reviewer 3**

Very nice revised manuscript. I find the current paper clear and think it is a valuable addition to the literature.
